# A Revision of the Traditional Analysis Method of Allometry to Allow Extension of the Normality-Borne Complexity of Error Structure: Examining the Adequacy of a Normal-Mixture Distribution-Driven Error Term

**DOI:** 10.1155/2022/8310213

**Published:** 2022-09-19

**Authors:** Enrique Villa-Diharce, Hector Alonso Echavarria-Heras, Abelardo Montesinos-López, Cecilia Leal-Ramírez

**Affiliations:** ^1^Centro de Investigación en Matemáticas, A.C. Jalisco S/N, Mineral Valenciana, Guanajuato, Gto 36240, Mexico; ^2^Centro de Investigación Científica y de Estudios Superiores de Ensenada, Carretera Ensenada-Tijuana No. 3918, Zona Playitas, 22860, 360 Ensenada, B.C., Mexico; ^3^Departamento de Matemáticas, Centro Universitario de Ciencias Exactas e Ingenierías (CUCEI), Universidad de Guadalajara, 44430 Guadalajara, Jalisco, Mexico

## Abstract

Huxley's model of simple allometry provides a parsimonious scheme for examining scaling relationships in scientific research, resource management, and species conservation endeavors. Factors including biological error, analysis method, sample size, and overall data quality can undermine the reliability of a fit of Huxley's model. Customary amendments enhance the complexity of the power function-conveyed systematic term while keeping the usual normality-borne error structure. The resulting protocols bear multiple-parameter complex allometry forms that could pose interpretative shortcomings and parameter estimation difficulties, and even being empirically pertinent, they could potentially bear overfitting. A subsequent heavy-tailed Q-Q normal spread often remains undetected since the adequacy of a normally distributed error term remains unexplored. Previously, we promoted the advantages of keeping Huxley's model-driven systematic part while switching to a logistically distributed error term to improve fit quality. Here, we analyzed eelgrass leaf biomass and area data exhibiting a marked size-related heterogeneity, perhaps explaining a lack of systematization at data gathering. Overdispersion precluded adequacy of the logistically adapted protocol, thereby suggesting processing data through a median absolute deviation scheme aimed to remove unduly replicates. Nevertheless, achieving regularity to Huxley's power function-like trend required the removal of many replicates, thereby questioning the integrity of a data cleaning approach. But, we managed to adapt the complexity of the error term to reliably identify Huxley's model-like systematic part masked by variability in data. Achieving this relied on an error term conforming to a normal mixture distribution which successfully managed overdispersion in data. Compared to normal-complex allometry and data cleaning composites present arrangement delivered a coherent Q-Q normal mixture spread and a remarkable reproducibility strength of derived proxies. By keeping the analysis within Huxley's original theory, the present approach enables substantiating nondestructive allometric proxies aimed at eelgrass conservation. The viewpoint endorsed here could also make data cleaning unnecessary.

## 1. Introduction

Julian Huxley envisioned the notion of constant relative growth between the size of a trait *y* and overall body size *x* [1–3]. Concurring formulation ordinarily referred to as Huxley's model of simple allometry is expressed through the power-function law:

(1) $$\begin{array}{c} y=\beta x^{\alpha }. \end{array}$$

The power function form bearing Huxley's model sustains both theoretical and empirical approaches in many research endeavors, e.g., biology [4–6], physics [7], economy [8], earth and atmospheric sciences [9], ecology [10], and resource management [11]. Allometric methods are particularly relevant in seagrass research. Seagrass species provide valuable ecological services in estuaries and nearshore environments, for instance, by offering food and shelter for a myriad of ecologically and economically valued marine organisms [12–14], contributing to nutrient cycling [15, 16], favoring the stabilization of the shoreline as roots and rhizomes compact the substrate, preventing erosion [17, 18], participating in the foundation of the detrital food web [19], and also playing a fundamental role in carbon sequestration [20]. In seagrass research, allometric methods mainly aim at predicting response to changing environmental conditions or analyzing growth patterns, for example, the relationship between the width of the leaves and their dry weight [21], the relationship between the length of stems and their density [22], and the relationship between the size of the leaves and their dry weight [23].

*Zostera marina L.* also known as eelgrass is an essential seagrass species providing vital ecological services in estuaries and nearshore environments. In addition to the aforelisted seagrass benefits, eelgrass offers a nursery for waterfowl and fish species and nutrient recycling. But despite the ecological relevance of eelgrass meadows, deleterious anthropogenic influences currently threaten their permanence [24]. Eelgrass remediation efforts mainly rely on transplanting endeavors [25]. Assessing the success of concurring plots depends on nondestructive estimations of standing stock from which total leaf biomass is an important constituent. When Huxley's model produces a reliable fit to an eelgrass leaf biomass and area data set, it could provide reliable surrogates of eelgrass leaf biomass based on direct nondestructive measurements of leaf area. Conceiving present allometric examination methods aim to enhance the efficiency of Huxley's model-based constructs for eelgrass conservation.

Despite the pertinence of Huxley's model, some factors limit the accuracy of deriving projections. Firstly, a response variable y expressed as a function of its covariate *x* through Equation (1) is extremely sensitive to the variation of estimates of the parameters *α* and *β* [26]. Then, error propagation could undermine the precision of Huxley's model-based projections of response values. Other prime influencers on the accuracy of estimates of parameters in Huxley's model are the analysis method, sample size, and data quality [27–29]. Mainly, envisioning a suitable analysis method to get estimates of parameters in Huxley's model relies primarily on detecting the implicit variation pattern in the original data scales. In some settings, the data spread displays a heteroscedastic pattern characterized as an increasing variation in the response's replicates concerning or relative to the predictive variable. The traditional approach to assembling a regression scheme involves Huxley's power function form as the systematic part and a multiplicative error term specified as a lognormally distributed random variable. The resultant method entails parameter identification through nonlinear regression in the direct scales of data. Concurrent to this approach concerns contemplating a logarithmic transformation that allows the analysis transference from natural scales into geometrical space. Their examination involves a regression model including a systematic linear part and an additive and normally distributed error term. The method completes by performing a back transformation step determining the identified form of Huxley's model of simple allometry in the direct scales. The last phase requires using a factor seeking to correct the bias of retransformation [30–32]. We will refer to this log transformation approach as the traditional analysis method of allometry (TAMA). Oppositely to a heteroscedastic spread pattern, data in the original scales could adapt to a homoscedastic dispersion outline. Accordingly, it may be pertinent to assume the appropriateness of a regression model composing Huxley's power function form at the systematic part along with an additive and normally distributed error term. Such a scheme precludes a log transformation step and sets nonlinear regression in the direct data scales as a necessary protocol for parameter identification tasks. So built regression model refers here as a direct nonlinear regression scheme (DNLR).

In allometric examination, when addressing the traditional multiplicative, log transformation, or direct nonlinear regression schemes, the essential task should simultaneously identify the systematic part and the error term in the associating regression protocol. The systematic part and the error term are of chief importance since the first determines the trend and the second, the dispersion pattern of the data. Nevertheless, usually, the virtual run concerns the identification of the systematic component of the model. Such a drive embodies attempts to improve goodness-of-fit by modifying Huxley's systematic part while keeping a normality-borne shape of the error term. Such a move conceives constructs referred to as complex allometry forms [33–35]. Moreover, commonly, concurrent examination attempts to carry out the normality of errors without verifying the assumptions of this model. Such a tactic somehow averts due attention to questioning the adequacy of the complexity of the assumed distribution of the error term. But Montesinos-López et al. [36] stirred away from this practice. They dealt with a data set including pairs of measurements of eelgrass leaf biomass and area. A TAMA fit resulted inconsistent. Montesinos-López et al.'s [36] amendment conformed to a systematic linear part but switched to a nonnormal distribution-brought error term. Moreover, Montesinos-López et al. [36] found that clinging to a logistic distribution-based try, not only lead to a consistent residual spread but also, remarkably improved the reproducibility strength of proxies for average leaf biomass derived from allometric projections established by a TAMA approach.

Here, we analyzed eelgrass leaf biomass and area data adapted from a sample originally reported in Echavarria-Heras et al. [37]. Compared to [36], present data exhibited overdispersion and marked size-related heterogeneity, perhaps explained by a lack of systematization in data gathering. Linked spread pattern precluded consistency of a TAMA fit. Moreover, the modification based on a logistic error term addressed in [36], fitted hereafter data cleaning procedures, also failed to produce consistent results. A normal mixture distribution provides a convenient model for data sets displaying high variability and heterogeneity. Accordingly, we conjectured that while analyzing present data, keeping the systematic linear term inherent to the TAMA approach but bearing a mixture of two normal distributions as a candidate for error term distribution could be suitable. Compared to composites including multiple parameter-complex allometry forms, normality of errors, and data cleaning procedures, the consistency of the present arrangement fitted in raw data delivered a notable reproducibility strength of proxies of eelgrass leaf biomass. Identifying the referred allometric relationship upon Huxley's power function model framework avoids complications from complex allometry forms during verifying parameter invariance. This feature is crucial for genuinely nondestructive assessments since previously fitted parameters could be used to get allometric projections of eelgrass leaf biomass values.

Because of difficulties tied to the implementation of complex allometry constructs, the present findings certainly enhance the perception of looking for the appropriateness of the error distribution as a mechanism to achieve a better fit of Huxley's model as suggested by Montesinos-López et al. [36]. Present findings exhibit the strength of a normal mixture distribution-borne error term as a device to produce a consistent fit of Huxley's model in a scenario of marked variability in data. It is also worth emphasizing that by keeping the analysis within the confines of Huxley's original theory, the current approach enables substantiating nondestructive allometric proxies aimed at eelgrass conservation. Besides, the scheme endorsed here could also make data cleaning unnecessary. And, since we provide a detailed explanation of the implementation, including mathematical, statistical, and computational aspects, the offered scheme can be straightforwardly adapted to other allometric examination endeavors. Also, our mixture distribution assumption on driving the error term bears a path yet not undertaken within the traditional analytical assortment of allometry. Therefore, we considered it worth reporting its suitability, and this manuscript devotes itself to that aim.

This work is structured as follows: In Section 2, we explain the formalities of the basic regression schemes deriving from Equation (1) and that circumscribe to a normal distribution-borne error term. For comparison aims in Section 3, we include the spread plots of the present data and that analyzed by Montesinos-López et al. [36]. Section 3 also elaborates on the necessary modifications of the basic regression schemes introduced in Section 2 that allow consideration of error terms driven by either a logistic or normal mixture distributions. Section 3 presents the results of conceived regression protocols fitted to the present data and compares their reproducibility power. The results section also incorporates a simulation study aimed to establish the strength of the approach under a known scenario.  Section 4 pertains to the discussion that stresses the strengths and weaknesses of the current approach. We strain on that by keeping allometric examination within the confines of Huxley's original theory; the present process bears advantages while substantiating nondestructive assessment proxies aimed at eelgrass conservation.  Section 5 presents the conclusions of this study and suggests future work.  Appendix A elaborates on reproducibility measures to assess the suitability of the allometric projection methods offered here.  Appendix B presents the formalities of the AIC index-based comparison of models fitted on different scales.

## 2. Materials and Methods

### 2.1. Huxley's Multiplicative Error Model

There are settings in allometric analysis with a spread in the original scales of data displaying a pattern of increasing variation in the response concerning itself or relative to the predictive variable. Assembling a candidate regression scheme usually undertakes a Huxley's multiplicative error model. It involves a systematic part acquiring a power function form and an error term specified as a function *δ*(*ϵ*) of a random variable *ϵ* that acts in a multiplicative way, namely,

(2) $$\begin{array}{c} y=\beta x^{\alpha }\delta \left(\epsilon \right), \end{array}$$

where *y* stands for the response variable, *x* for the covariate, *α* and *β* are parameters, *δ*(*ϵ*) = exp(*ϵ*) and *ϵ* taken as a normally distributed random variable having zero mean and deviation *σ*; that is, *ϵ* ~ *N*(0, *σ*) [38–40]. Proposed form sets *δ*(*ϵ*) as a lognormally distributed random variable with zero log-mean and log-deviation *σ*, that is, *δ*(*ϵ*) ~ lognorm(0, *σ*). The likelihood function takes the form

(3) $$\begin{array}{c} L\left(\beta ,\alpha ,\sigma \right)=\overset{n}{\underset{i=1}{\prod }}\left\{\frac{1}{\sqrt{2\pi \sigma^{2}}y_{i}}\exp\left[-\frac{1}{2\sigma^{2}}{\left(\log\left(y_{i}\right)-\mu_{i}\right)}^{2}\right]\right\}, \end{array}$$

where *μ*_*i*_ = log(*β*) + *α*log(*a*_*i*_).

Besides, as it is set by Equation (2), the variability of the response at a given value of the covariate regulates by the contribution of the random error *δ*(*ϵ*) and the value of the systematic part *βx*^*α*^. So, for large covariate values, the named range of variation grows, thus resulting in a heteroscedastic statistical model. For present aims, the model set by Equation (2) refers as Huxley's multiplicative error model (HMEM) or simply as a MEM protocol.

Assuming suitability of the model of Equation (2), we usually address the problem of acquiring the mean of the response *y*  conditioned on a covariate value *x*. Associating form denoting here through *E*(*y*|*x*) is gotten by taking the expected value on both sides of Equation (2) conditioned by the explanatory variable *x*, namely,

(4) $$\begin{array}{c} E\left(y\left|x\right.\right)=\beta x^{\alpha }E\left(\delta \right) \end{array}$$

where

(5) $$\begin{array}{c} E\left(\delta \right)=\exp\left(\frac{\sigma^{2}}{2}\right). \end{array}$$

Then, to obtain the mean response *E*(*y*|*x*), in addition to the power function-like systematic term *βx*^*α*^, we must consider a factor *E*(*δ*), which interprets as a correction factor (CF) for bias of allometric projection of the mean response through the estimated form of the power function *βx*^*α*^. Only in the case of Huxley's lognormal multiplicative error model the correction factor *E*(*δ*) takes on the form given by Equation (4). In the general settings given the distribution of the random variable *δ*(*ϵ*) we could attempt to obtain a closed-form for the correction factor *E*(*δ*) by evaluating the expectation of the response variable *y*. It is worth emphasizing that in getting a closed form for *E*(*δ*), it is essential to identify the form of the distribution acquired by the error term *δ*(*ϵ*).

### 2.2. The Traditional Analysis Method of Allometry

Concurrent to Huxley's lognormal multiplicative error model of Equation (2), there is an approach relying on a log transformation: (*x*, *y*)⟶(*u*, *v*) = (*lnx*, *lny*), that allows the contemplation of a linear regression model in the geometrical scales, namely,

(6) $$\begin{array}{c} v=\beta_{0}+\alpha u+\epsilon , \end{array}$$

where *β*_0_ = *lnβ* and with an additive error term *ϵ* = ln(*δ*) expressing as a normally distributed random variable having zero mean and deviation *σ*, that is, *ϵ* ~ *N*(0, *σ*). The likelihood function is as follows:

(7) $$\begin{array}{c} L\left(\beta ,\alpha ,\sigma \right)=\overset{n}{\underset{i=1}{\prod }}\left(\frac{1}{\sqrt{2\pi }\sigma }\right)\exp\left\{-\frac{1}{2}{\left(\frac{v_{i}-\mu_{i}}{\sigma }\right)}^{2}\right\}, \end{array}$$

and with *μ*_*i*_ = *β*_0_ + *αu*_*i*_.

Based on the identified form of Equation (6), we perform a back transformation step to get estimated form of the mean response function of Equation (4) and that of the correction factor (5) [30–32]. Afterwards, we refer to the protocol of Equation (4) as the traditional analysis method of allometry (TAMA) [41–43].

### 2.3. The Direct Nonlinear Regression Protocol

Oppositely to a circumstance described by the Huxley's lognormal multiplicative error model of Equation (2), it may be pertinent to conceive a regression model where the systematic power function-like term maintains, but that the random error contributes additively to the variability of the response *y*, that is,

(8) $$\begin{array}{c} y=\beta x^{\alpha }+\epsilon , \end{array}$$

with *ϵ* usually assumed as a normally distributed random variable having a zero mean and a deviation *σ*, that is, *ϵ* ~ *N*(0, *σ*). Therefore, oppositely to the heteroscedastic spread entailed by the multiplicative error model of Equation (2) for the additive error model of Equation (6), the contribution of *ϵ* to *y* variability is its value itself, being this null when *ϵ* vanishes. The likelihood function turns out to be

(9) $$\begin{array}{c} L\left(\beta ,\alpha ,\sigma \right)=\overset{n}{\underset{i=1}{\prod }}\left(\frac{1}{\sqrt{2\pi }\sigma }\right)\exp\left\{-\frac{1}{2}{\left(\frac{y_{i}-\mu_{i}}{\sigma }\right)}^{2}\right\}, \end{array}$$

with *μ*_*i*_ = *βx*_*i*_^*α*^.

We further on refer to the scheme of Equation (6) as direct nonlinear regression (DNLR) [40, 44–46].

In what follows, we will refer generically to the error structure of a given regression scheme as a merge of the way the error term enters into the model and the distribution that drives its stochasticity. Accordingly, we say that the model of Equation (2) bears a multiplicative-lognormal error structure and that the models of Equation (6) and Equation (8) both share an additive-normal error structure. For the aim of exploring the extent of modifying the error structure of the MEM, TAMA, or DNLR schemes in what follows, we conceive composite regression schemes that maintain the involved systematic terms but modify the assumption on the error-shaping random variable *ϵ* from normality to being logistically distributed or else, according to a mixture of two normal distributions of common zero mean but different deviations. Particularly, for the DNLR scheme we adapt a Breusch-Pagan [47] type variance function form, aimed to take over heteroscedasticity. For comparison, we include the polynomial modification to TAMA's scheme undertaken by Echavarría-Heras et al. [37]. Formal expressions of the composite regression schemes addressed here appear in the results section.

## 3. Results

### 3.1. Data

**Figure 1:**
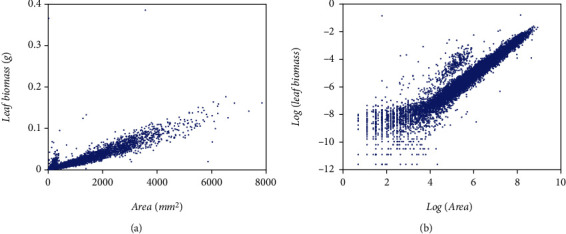
Spread diagrams of eelgrass leaf weight [g] against relating area [mm^2^] composing the 13 months sampling data set arranged by Echavarría-Heras et al. [37]. (a) Dispersion on the arithmetical scales. (b) Dispersion in log scales. We can be aware of extreme variability of replicates as well of marked heterogeneity of spread patterns for smaller and larger leaf area values. This allocates spread in the geometrical space.

Present examination relies in a data set comprising pairs of measurements of leaf weight *y* [g] and relating area *x* [mm^2^], adapted from a sample reported in Echavarría Heras et al. [37] obtained by a 13-month sampling performed on an eelgrass meadow in Ensenada, B.C., Mexico.  Figure 1(a) displays data spread in the original arithmetical scales. We can be aware of noticeable variability of replicates as well as of marked heterogeneity of patterns among the pools of smaller and larger leaf area values in the sample.  Figure 1(b) pertains to spread corresponding to log scales. To compose present data set we removed two of the 10412 pairs reported. We further refer to the resulting 10,410 pairs as the present data set that could be also indistinctly referred by means of the symbol EHDS, for Echavarría-Heras et al. [37] data set.

**Figure 2:**
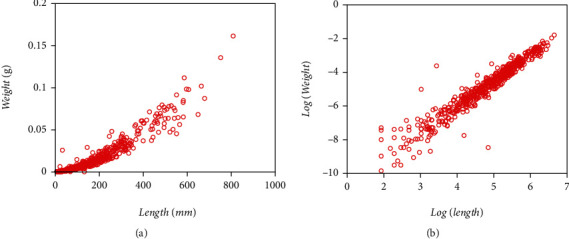
Spread diagrams of eelgrass leaf weight [g] against relating area [mm^2^] the data set addressed by Montesinos-López et al. [36]. (a) Dispersion on the arithmetical scales. (b) A spread on the geometrical space.

For comparison aims, we depend on a second eelgrass leaf biomass to area data set examined by Montesinos-López et al. [36] and collected at the same meadow as the EHDS. It composes a total of 537 pairs of measurements of leaf weight (*y*) and relating area (*x*), also gathered in the Echavarria-Heras et al. [37] study site but only over a one-month sampling. Ahead this data set identifies by the label MLDS, for Montesinos-López et al. [36] data set.  Figure 2(a) displays associating spread in the original arithmetical scales.  Figure 2(b) presents that one corresponding to geometrical scales.

### 3.2. Performance of the MEM and TAMA Schemes

**Figure 3:**
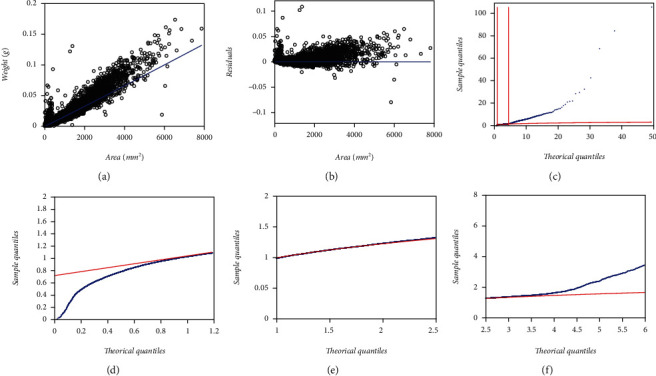
Spread plots of the fit of a MEM scheme as given by Equations (2) and (3) on present data (EHDS). Uppermost panels display the diagrams of dispersion about the fitted systematic part, residuals, and Q-Q lognormal plot. The lowermost panels exhibit a close-up split of the different regions associated with the Q-Q lognormal diagram. (a) Dispersion around MEM-fitted power function in direct scales. (b) The scatter diagram of residuals against the area values. (c) The Q-Q lognormal plot of the residuals on the direct scales. Vertical lines in (c) delimit the linear part of the Q-Q lognormal plot, placing in the interval (0.240, 3.883), between the ordered observations, num. 700 and the 9500 of the 10410 original data pairs. (e) The linear sector. (d, f) The pattern at the extremes departs from that corresponding to a lognormal distribution; namely, we have heavy tails in the set of residuals corresponding to the MEM's fit.

**Figure 4:**
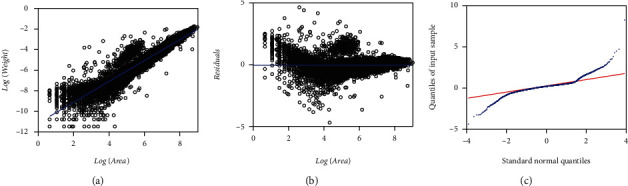
Dispersions about the fitted systematic part, residuals, and Q-Q diagram of a TAMA scheme as given by Equations (6) and (7) and fitted on the Echavarría-Heras et al. [37] data. (a) Dispersion around TAMA-fitted line in log scales. (b) The scatter diagram of residuals against the logarithm of the area. (c) The Q-Q normal plot of the residuals on a geometric scale. Heavy tails refer to an unreliable TAMA fit.

The heteroscedastic pattern shown in Figure 1(a) suggests exploring the suitability of a MEM assumption. Therefore, we could firstly analyze the EHDS according to the regression model of Equations (2) and (3) or equivalently by calling on to the concurring TAMA scheme in geometrical scales appointed by Equations (6) and (7). Including this last fit provides insight at envisioning the actual distribution of the error term contemplated in the regular MEM scheme. Also incorporating the TAMA-based fits allows comparing present findings to results reported by Echavarría-Heras et al. [37], as well as to those by Montesinos-López et al. [36] when analyzing the MLDS.  Table 1 presents fitting statistics of a MEM try, and those relating to a TAMA fit do in Table 2. Spread plots on both the MEM and the TAMA fits appear in Figures 3 and 4 one to one.  Figure 3(a) displays the spread about the fitted MEM's power function-like systematic part.  Figure 3(b) suggests that the error term does not match the expected lognormal distribution pattern. Besides, Figure 3(c) shows the associating Q-Q lognormal plot of the residuals. Vertical lines sketched in Figure 3(c) delimit the linear part of the Q-Q lognormal plot. Such a sector places in the interval (0.240, 3.883), between the ordered observations, num. 700 and the 9500 of the 10410 original data pairs. Lowermost panels in Figure 3 display a close-up split of the Q-Q lognormal diagram in Figure 3(c).  Figure 3(e) associates with the linear sector. Figures 3(d) and 3(f) reveal that we have heavy tails in the set of residuals corresponding to a MEM's fit on the EHDS.

Correspondingly, Figure 4(a) shows the spread about the TAMA's linear systematic part (cf. Equations (6) and (7) fitted to the EHDS in geometrical scales. Moreover, Figure 4(b) already suggest that the error term does not match the expected normal distribution pattern. Besides, opposing to a masked heavy tail at the left extreme of Figure 3(c) the spread in Figure 4(c) clearly reveals that while the normal distribution fits adequately in the central part, the pattern at the extremes departs from that corresponding to a normal distribution; namely, we have heavy tails in TAMA's set of residuals. Concerning this fit, calculating the value of the kurtosis coefficient provides additional evidence of the existence of heavy tails in the distribution of the error term. Certainly, the associating kurtosis coefficient attained a value of kurt = 14,176, which is a much larger value than the one corresponding to a normal distribution (kurt = 3.0).According to Wheeler [48], such a big value indicates that the distribution of the residues has heavy tails since kurtosis provides information on the extremes rather than the central part of the distribution. Therefore, a TAMA scheme turns out to be very simple to produce a reliable fit on the EHDS. Indeed, the analyzed data exhibit a variability pattern suggesting that the complexity of an error structure beyond that one bearing to normality turns out to be necessary to grant a coherent fit. Thus, the resulting residuals should instead model through a distribution with a considerably greater overdispersion than the normal one in the geometric scales could explain. In summary, results suggest that a normal distribution assumption for the random variable *ϵ* inherent to the MEM and TAMA fits does not support a suitable model for the variability pattern inherent to the EHDS.

### 3.3. Implementation of the Breusch-Pagan Modification on the Regular DNLR Protocol

As it conceives here, a DLNR-BP protocol stands for a Breusch-Pagan [47] modification of the basic DLNR scheme of Equation (6) envisioned to account for the heteroscedastic pattern shown in Figure 1(a). Formally, a DNLR-BP adaptation acquires a form:

(10) $$\begin{array}{c} y=\beta x^{\alpha }+\in \end{array}$$

with *ϵ*  taken as normally distributed random variable, having a zero mean, and a covariate dependent deviation *σ*(*x*), that is, *ϵ* ~ *N*(0, *σ*(*x*)). To offer a suitable candidate form for *σ*(*x*), we recall the procedure yielding the Breusch-Pagan [47] test, so we set the following:

(11) $$\begin{array}{c} \sigma \left(y\left|x\right.\right)=\sigma \left(1+kx\right) \end{array}$$

Additionally, the likelihood function becomes the following:

(12) $$\begin{array}{c} L\left(\beta ,\alpha ,\text{k},\sigma \right)=\overset{n}{\underset{i=1}{\prod }}\left(\frac{1}{\sqrt{2\pi \sigma \left(y_{i}\left|x_{i}\right.\right)}}\right)\exp\left\{-\frac{1}{2}{\left(\frac{y_{i}-\mu_{i}}{\sigma \left(y_{i}\left|x_{i}\right.\right)}\right)}^{2}\right\}, \end{array}$$

with *μ*_*i*_ = *βx*_*i*_^*α*^ and *σ*(*y*_*i*_|*x*_*i*_) = *σ*(1 + *kx*_*i*_).

**Figure 5:**
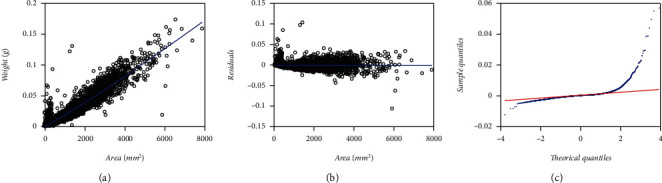
Spreads about mean response curve, that of residual and the one portraying in the Q-Q normal plot of the heteroscedastic DNLR-BP scheme of Equations (10) and (12) fitted on the Echavarría-Heras et al. [37] data set. (a) Dispersion about fitted Huxley's power function form. (b) The scatter diagram of residuals against leaf area. (c) The Q-Q normal plot of the residuals on direct arithmetical scales. Heavy tails appear through a markedly asymmetrical pattern which refers to an overall inconsistent DNLR-BP fit.

Table 3 presents parameter estimates and associating fitting statistics of the DNLR-BP scheme of Equations (10) and (12) performing on the EHDS. The resulting spread plots appear in Figure 5. In Figure 5(a), we exhibit the dispersion about fitted Huxley's power function form.  Figure 5(b) shows the scatter diagram of residuals against leaf area. In turn, Figure 5(c) shows a Q-Q normal plot of the residuals on direct arithmetical scales. Although spreads in (a) and (b) suggest the presence of two correspondence rules conforming to the global area-weight relationship, the fitted power function deems roughly consistent. But still, Figure 5(c) displays a heavy tails pattern of the error term, although now being asymmetrical. Such a Q-Q normal diagram perhaps explains by the fact that even by appointing a Breusch-Pagan [47] variance function form, that move failed to provide a sound model for the heterogeneity of the heteroscedastic spread. But said asymmetrical spread could additionally explain by the Lai et al. [40] observation that direct nonlinear regression can produce bias for large covariate values. Moreover, comparison of AIC values among the MEM (AIC = −94745.76) and the DNLR-BP (AIC = −84528.9) fit produces ΔAIC = −10217, favoring the MEM fit. Therefore, a multiplicative error structure seems more fit at analyzing the EHDS. The relatively fair spread about the fitted systematic term hints on adequacy of Huxley's model at describing the trend in the EHDS but the spreads in the Q-Q normal diagrams accompanying the MEM, TAMA, and DNLR-BP fits suggest that assuming the error shaping random variable *ϵ* as being normally distributed is statistically unsupported.

### 3.4. Execution of the Logistic Distribution Amendments to the Regular MEM and TAMA Schemes

Agreeing to Montesinos-López et al. [36], while analyzing the EHDS, we attempted to model overdispersion or residuals produced by the regular MEM or TAMA fits by agreeing to a complexity of error structure adaptation approach. We assumed first that the basic random variable *ϵ* better conforms to a logistic distribution, since this last one works fine when the overdispersion is low. Resulting analytical arrangements describe by the acronyms MEM-loglogistic and TAMA-logistic. Formally, the MEM-loglogistic construct stands for a modification of the regression model of Equation (2), which establishes through

(13) $$\begin{array}{c} y=\beta x^{\alpha }\delta \left(\in \right) \end{array}$$

with *δ*(*ϵ*) = exp(*ϵ*) and *ϵ* taken as a logistically distributed random variable having zero location and scale  *σ*, that is, *ϵ* ~ logistic (0, *σ*). Proposed form sets *δ*(*ϵ*) as a loglogistically distributed random variable with zero log-location and log-scale *σ*. The likelihood function takes a form:

(14) $$\begin{array}{c} L\left(\beta ,\alpha ,\sigma \right)=\overset{n}{\underset{i=1}{\prod }}\frac{\exp\left[\left(\ln\left(y_{i}\right)-\ln\left(\mu_{i}\right)\right)/\sigma \right]}{\sigma {\left[1+\exp\left[\left(\ln\left(y_{i}\right)-\ln\left(\mu_{i}\right)\right)/\sigma \right]\right]}^{2}}, \end{array}$$

being *μ*_*i*_ = *βa*_*i*_^*α*^. The mean response function becomes the following:

(15) $$\begin{array}{c} E\left(y\left|x\right.\right)=\beta x^{\alpha }E\left(\delta \right), \end{array}$$

where

(16) $$\begin{array}{c} E\left(\delta \right)=\Gamma \left(1+\sigma \right)\Gamma \left(1-\sigma \right). \end{array}$$

Meanwhile, according to Equation (5), a TAMA-logistic scheme sets by the model

(17) $$\begin{array}{c} v=\beta +\alpha u+\epsilon , \end{array}$$

with *ϵ* ~ logistic(0, *σ*). The likelihood function takes on a form:

(18) $$\begin{array}{c} L\left(\beta ,\alpha ,\sigma \right)=\overset{n}{\underset{i=1}{\prod }}\frac{\exp\left(\left(v_{i}-\mu_{i}\right)/\sigma \right)}{\sigma {\left[1+\exp\left(\left(v_{i}-\mu_{i}\right)/\sigma \right)\right]}^{2}}, \end{array}$$

where *μ*_*i*_ = *β*_0_ + *αu*_*i*_. The back transformation step requires using the correction factor

(19) $$\begin{array}{c} \text{CF}=\Gamma \left(1+\sigma \right)\Gamma \left(1-\sigma \right). \end{array}$$

**Figure 6:**
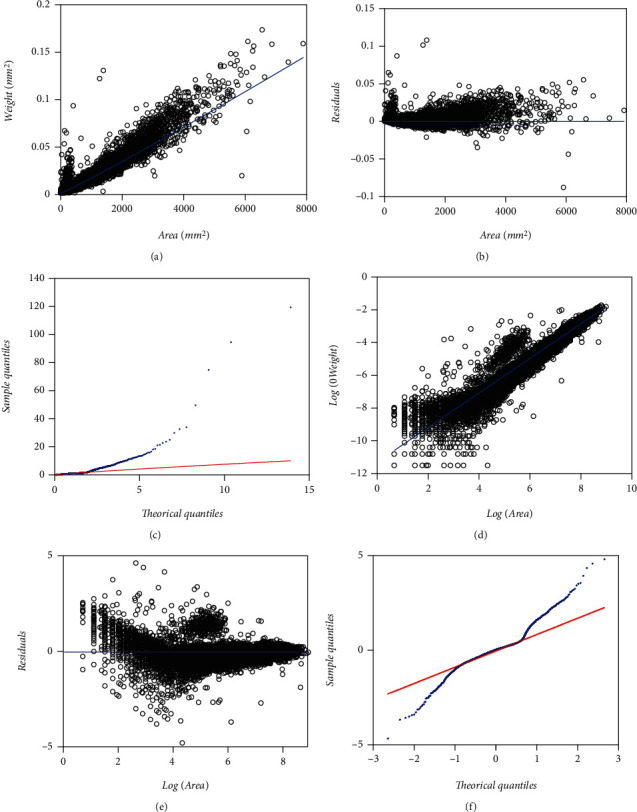
Fitted systematic part residual and Q-Q plots of a MEM-loglogistic (cf. Equations (13) and (14)) and a TAMA-logistic (cf. Equations (17) and (18)) fitted on the Echavarría-Heras et al. [37] data. (a) Dispersion about the MEM-loglogistic fitted Huxley's power function systematic part. (b) Resulting MEM-loglogistic residual spread and (c) presents corresponding heavy tails displaying in Q-Q loglogistic diagram. Correspondingly, (d) displays dispersion around the TAMA-logistic line in log scales. (e) The scatter diagram of residuals against the logarithm of leaf area. (f) The Q-Q logistic plot of the residuals on geometrical space. Heavy tails refer to an inconsistent TAMA-logistic fit.

Estimated parameter values and relating statistics for a MEM-loglogistic protocol of Equations (13) and (14) fitted on the EHDS appear in Table 4. Correspondingly, those pertaining to a TAMA-logistic fit (cf. Equations (17) and (18)) display in Table 5. In Figure 6, we include spread plots on both the MEM-loglogistic and the TAMA-logistic fits. Besides, upper panels of Figure 6 present spreads associating with MEM-loglogistic fit, that is, Figure 6(a) includes spread about fitted Huxley's power function, Figure 6(b) corresponding to residual's dispersion and Figure 6(c) devoting to the Q-Q loglogistic diagram spread of residuals in direct arithmetical scales. As we arranged for Figure 3(c), by splitting the diagram in Figure 6(c) and then amplifying we can also be aware of that the pattern at the extremes departs from that corresponding to a loglogistic distribution; namely, we have heavy tails in the set of residuals corresponding to the MEM-loglogistic fit. Correspondingly, lower panels in Figure 6 present dispersion patterns resulting from a TAMA-logistic fit.  Figure 6(d) portraits dispersion about fitted TAMA-logistic line.  Figure 6(e) exhibits residual dispersion led by the TAMA-logistic fit. And Figure 6(f) portraits the corresponding Q-Q logistic plot of residuals in geometrical space. Again, a heavy tails pattern shows. For the sake of conciseness when comparing to the Montesinos-López et al. [36] fit, we only discuss the implications of the TAMA-logistic spread plots. Compared to a regular TAMA fit present residual spread still shows an uneven pattern. Moreover, the improvement in consistency of the Q-Q logistic plot spread reported by Montesinos-López et al. [36] did not show up in present EHDS fit. Indeed, contrasted to the Q-Q logistic plot spread shown in Montesinos-López et al. [36] the Q-Q logistic plot in Figure 6(f) still stands to a heavy tails pattern. Therefore, in contraposition to the results reported by Montesinos-López et al. [36] the present TAMA-logistic scheme turned on unsuccessful at modeling the allometric relationship intrinsic to the EHDS.

The logistic distribution can reasonably model a higher dispersion than the normal one [36]. But the heavy tails arrangement displaying in the Q-Q loglogistic diagram of Figure 6(c) refers to an unfair MEM-loglogistic fit (Equation (9)), and equally, the Q-Q logistic plot of the residuals of a TAMA-logistic fit (Figure 6(f)) on geometrical space showing heavy tails also point to an inconsistent TAMA-logistic (Equation (10)) fit.

### 3.5. Performing the Breusch-Pagan and Logistic Distributed Error Modification on the DNLR Protocol

As it conceives here, a DLNR-BP-logistic protocol stands for a Breusch-Pagan [47] along with a logistically distributed error modification of the basic DLNR scheme of Equation (6) envisioned to account for the heteroscedastic pattern shown in Figure 1(a). Formally, a DNLR-BP-logistic adaptation admits a form:

(20) $$\begin{array}{c} y=\beta x^{\alpha }+\in \end{array}$$

with *ϵ*  taken as a logistically distributed random variable, having a zero location and a covariate dependent scale *σ*(*x*), that is, *ϵ* ~ logistic(0, *σ*(*x*)), where we set the following:

(21) $$\begin{array}{c} \sigma \left(y\left|x\right.\right)=\sigma \left(1+kx\right) \end{array}$$

The likelihood function becomes

(22) $$\begin{array}{c} L\left(\beta ,\alpha ,\sigma \right)=\overset{n}{\underset{i=1}{\prod }}\frac{\exp\left(\left(y_{i}-\mu_{i}\right)/\sigma_{i}\right)\;}{\sigma_{i}{\left[1+\exp\left(\left(y_{i}-\mu_{i}\right)/\sigma_{i}\right)\;\right]}^{2}}, \end{array}$$

with *μ*_*i*_ = *βx*_*i*_^*α*^, and *σ*_*i*_ = *σ*(1 + *kx*_*i*_).

**Figure 7:**
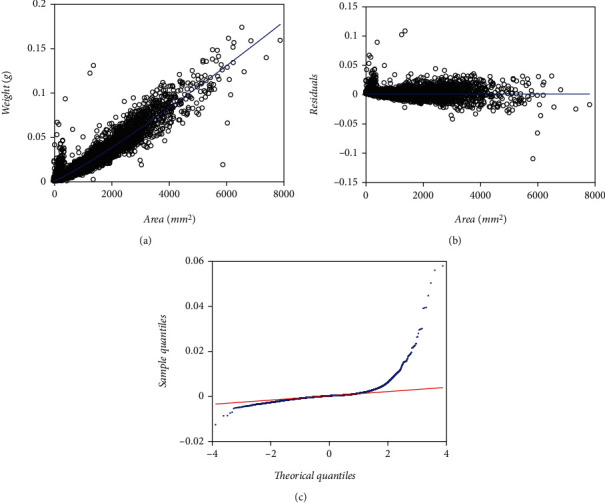
Spreads about mean response curve that of residual and the one portraying in the Q-Q logistic plot of the heteroscedastic DNLR-BP-logistic scheme of Equations (20) and (22) fitted on the Echavarría-Heras et al. [37] data set. (a) Dispersion about fitted Huxley's power function form. (b) The scatter diagram of residuals against leaf area. (c) The Q-Q logistic plot of the residuals on direct arithmetical scales. Heavy tails appear through a markedly asymmetrical pattern which refers to an overall inconsistent DNLR-BP-logistic fit.

Table 6 presents parameter estimates and associating fitting statistics of the DNLR-BP-logistic scheme as given by Equations (20) and (22) and performing on the EHDS. The resulting spread plots appear in Figure 7. In Figure 7(a), we exhibit the dispersion about fitted Huxley's power function form.  Figure 7(b) shows the scatter diagram of residuals against leaf area. In turn, Figure 7(c) shows a Q-Q logistic plot of the residuals on direct arithmetical scales. Moreover, comparison of AIC values among the MEM-logistic (AIC = −97297.3) and the DNLR-BP-logistic (AIC = −84528.9) fits produces ΔAIC = −12768, favoring the MEM-logistic protocol, with an AIC difference that widens over that recorded for a comparison between the MEM and DNLR-BP schemes. And yet, Figure 7(c) displays a heavy tails pattern of the error term, once again being asymmetrical as in the case of a DNLR-BP fit. Again, such an asymmetrical spread could explain by the embedding of a real multiplicative error structure into an additive error counterpart. An on top of that, the Lai et al. [40] observation of biased direct nonlinear regression output for large covariate values could be also pertinent. In any event, the spread in the Q-Q logistic in Figure 7(c) bears that assuming logistically distributed residuals turns out to be unfeasible. At this point of the matter, by looking at a relatively fair spread about the systematic parts of fitted MEM, TAMA, DNLR-BP schemes and their subsequent modifications to contemplate a logistic distribution-borne error term, it is not idle to say that the detected inconsistencies in Q-Q diagram spreads, could already hint at a lack of suitability of either a normal or a logistic distribution as compatible models for the residual dispersion in present data.

### 3.6. Implementation of the TAMA Scheme Fitted on Processed Data

**Figure 8:**
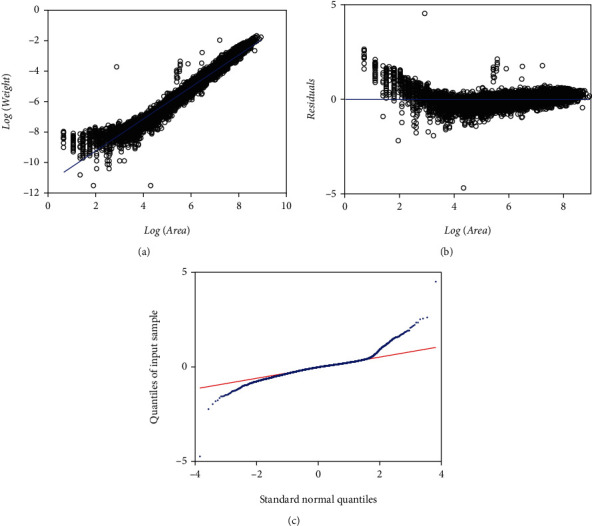
Spreads about mean response curve, that of residual and the one portraying in the Q-Q normal plot of a TAMA scheme of Equations (6) and (7) fitted on the data set produced by applying the mean absolute deviation data cleaning procedures described in Echavarría-Heras et al. [37] on present data. (a) Dispersion about fitted Huxley's linear systematic part. (b) The scatter diagram of residuals against leaf area. (c) The Q-Q normal plot of the residuals that shows heavier tails than those expected for a normal distribution.

Heterogeneity of spread in Figure 1 could perhaps explain by the participation of multiple agents that contributed to data gathering without standardization of routines [37]. Then, at first glance, proliferation of unduly replicates could generate issues at data quality that elucidate the lack of fit of schemes based on Huxley's model and accompanying normal or logistic distributions-borne error terms. Echavarria-Heras et al. [28] adapted a median absolute deviation procedure to remove anomalous replicates in a sample of similar allometric eelgrass data. We engaged these procedures on present data to explore the extent of data quality influences in determining the inconsistencies of the fits above.  Table 7 presents parameter estimates and related statistics associating with a TAMA scheme as given by Equations (6) and (7) and fitted on the processed EHDS. These statistics on the processed data are only provided for completion of the presentation and do not intend to support comparison to the remaining fits as those relied upon the crude EHDS.  Figure 8 displays the spread plots associated with a TAMA fit on processed data.  Figure 8(a) displays dispersion about fitted Huxley's linear systematic part.  Figure 8(b) shows the scatter diagram of residuals against leaf area.  Figure 8(c) presents the Q-Q normal plot of the residuals that shows heavier tails than those expected for a normal distribution. Even though the addressed median absolute deviation procedure removed a large share (25%) of the original data, the TAMA fit could not normalize the residual dispersion. Moreover, differentiation of spread patterns among the pools' smaller and larger leaf sizes barely hinted by plots in Figure 1 seems to portray undoubtedly once data processing completes, as shown by (a) and (b) in Figure 8. Therefore, considering this and for improving the quality of fits of schemes involving Huxley's model-driven systematic term, it seems reasonably calling in distributions that allow a greater dispersion than the normal or the logistic ones at assembling the accompanying error term.

### 3.7. Assessments of the Normal Mixture Amendments to the Regular MEM and TAMA Schemes

Constituents in the family of finite mixtures of distributions are highly flexible due to the diversity of forms that they can acquire. Particularly, the distributions that we can construct through finite mixtures of normal distributions are very varied since we can obtain multimodal, skewed, and distributions with excess kurtosis. Particularly, at adapting the symmetric distribution with zero mean and heavy tails suggested by the spread in Figure 4(c), we could explore the appropriateness of a mixture of two individual normal distributions; *N*_1_(0, *σ*_1_) and *N*_2_(0, *σ*_2_) having a common zero mean but different deviations *σ*_1_and *σ*_2_.We assume also, that the weight through which *N*_1_(0, *σ*_1_) and *N*_2_(0, *σ*_2_) participate in the mixture designates by means of *p*. For simplicity further on we employ the symbol M2N to designate so conceived mixture distribution. Assume that the error shaping random variable *ϵ* in Equation (9) distributes according to a M2N mixture. Then, denoting associating probability density function through the symbol *f*(*ϵ*; *p*, *σ*_1_, *σ*_2_), we have the following:

(23) $$\begin{array}{c} f\left(\epsilon ;p,\sigma_{1},\sigma_{2}\right)=pf_{1}\left(\epsilon ;0,\sigma_{1}\right)+\left(1-p\right)f_{2}\left(\epsilon ;0,\sigma_{2}\right), \end{array}$$

where *f*_1_(*ϵ*; 0, *σ*_1_) and *f*_2_(*ϵ*; 0, *σ*_2_) stand for one to one the density functions of the components *N*_1_(0, *σ*_1_) and *N*_2_(0, *σ*_2_). It follows, directly from Equation (14) that *E*(*ϵ*) = 0  and that joining variance *σ*^2^(*ϵ*) takes on the form:

(24) $$\begin{array}{c} \sigma^{2}\left(\epsilon \right)=p\sigma_{1}^{2}+\left(1-p\right)\sigma_{2}^{2}. \end{array}$$

Therefore, the assumption of *ϵ* being distributed according to presently conceived normal mixture M2N symbolizes through *ϵ* ~ M2N(*p*, 0, *σ*) where *σ* derives from Equation (16). Whenever *ϵ* ~ M2N(*p*, 0, *σ*), the random variable *δ*(*ϵ*) = exp(*ϵ*) acquires a log-mixture of two normal distribution of zero log-mean, log-deviation *σ* and weight *p*, that is, *δ*(*ϵ*) ~ LM2N(*p*, 0, *σ*). Moreover, the associating density function denoted through *f*_*δ*_(*δ*; 0, *p*, *σ*_1,_*σ*_2_) is given by the following:

(25) $$\begin{array}{c} f_{\delta }\left(\delta ;0,p,\sigma_{1},\sigma_{2}\right)=pf_{1}\left(\delta ;0,\sigma_{1}\right)+\left(1-p\right)f_{2}\left(\delta ;0,\sigma_{2}\right), \end{array}$$

where *f*_1_(*δ*; 0, *σ*_1_) and *f*_2_(*δ*; 0, *σ*_2_) are the two lognormal density functions, with common log-mean zero and log-deviations *σ*_1_ and *σ*_2_, respectively.

Correspondingly, the adaptation of the MEM protocol that replaces a lognormally distributed error term by a LM2N alternate denotes by MEM-LogM2N. Formally, such a scheme takes on a form:

(26) $$\begin{array}{c} y=\beta x^{\alpha }\delta \left(\in \right) \end{array}$$

with *δ*(*ϵ*) = exp(*ϵ*) and *ϵ* taken as a M2N distributed random variable, that is, *ϵ* ~ M2N(*p*, 0, *σ*). Therefore, *δ*(*ϵ*) ~ LogM2N(0, *σ*). The likelihood function becomes the following:

(27) $$\begin{array}{c} L\left(\beta ,\alpha ,\sigma_{1},\sigma_{2},p\right)=\overset{n}{\underset{i=1}{\prod }}\left\{p\frac{1}{\sqrt{2\pi \sigma_{1}^{2}}y_{i}}\exp\left[-\frac{1}{2\sigma_{1}^{2}}{\left(\log\left(y_{i}\right)-\mu_{i}\right)}^{2}\right]+\left(1-p\right)\frac{1}{\sqrt{2\pi \sigma_{2}^{2}}y_{i}}\exp\left[-\frac{1}{2\sigma_{2}^{2}}{\left(\log\left(y_{i}\right)-\mu_{i}\right)}^{2}\right]\right\}, \end{array}$$

where *μ*_*i*_ = log(*β*) + *α*log(*x*_*i*_). And the mean response function takes on a form:

(28) $$\begin{array}{c} E\left(y\left|x\right.\right)=\beta x^{\alpha }E\left(\delta \right). \end{array}$$

Then, Equation (18) implies the following:

(29) $$\begin{array}{c} E\left(\delta \right)=p\exp\left(\frac{\sigma_{1}^{2}}{2}\right)+\left(1-p\right)\exp\left(\frac{\sigma_{2}^{2}}{2}\right). \end{array}$$

Besides, we use a composite TAMA-M2N to distinguish the regression arrangement that modifies the regular TAMA scheme to consider a M2N distributed error term. Formally, the scheme stands for a modification of the regression model of Equation (5) that establishes through the following:

(30) $$\begin{array}{c} v=\beta +\alpha u+\epsilon , \end{array}$$

with *ϵ* being a M2N distributed random variable with zero mean and deviation *σ*, that is, *ϵ* ~ M2N( 0, *σ*). The corresponding likelihood function is given by the following:

(31) $$\begin{array}{c} L\left(\beta_{0},\alpha ,\sigma_{1},\sigma_{2},p\right)=\overset{n}{\underset{i=1}{\prod }}\left\{p\frac{1}{\sqrt{2\pi \sigma_{1}^{2}}}\exp\left[-\frac{1}{2\sigma_{1}^{2}}{\left(v_{i}-\mu_{i}\right)}^{2}\right]+\left(1-p\right)\frac{1}{\sqrt{2\pi \sigma_{2}^{2}}}\exp\left[-\frac{1}{2\sigma_{2}^{2}}{\left(v_{i}-\mu_{i}\right)}^{2}\right]\right\}, \end{array}$$

where *μ*_*i*_ = *β*_0_ + *αu*_*i*_.

Correspondingly, according to Equation (19), the correction factor for retransformation aims becomes the following:

(32) $$\begin{array}{c} CF=p\exp\left(\sigma_{1}^{2}/2\right)+\left(1-p\right)\exp\left(\sigma_{2}^{2}/2\right) \end{array}$$

**Figure 9:**
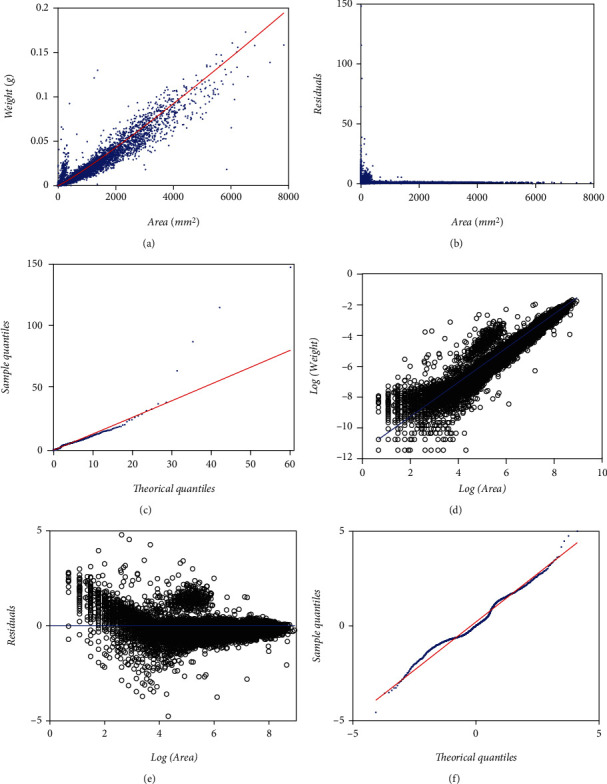
Spread plots relating to a M2N-driven modification to the MEM (cf. Equations (26) and (27)) (upper panels) and those corresponding to a TAMA-M2N composite (cf. Equations (30) and (31)) both fitted on the EHDS. (a) Dispersion around fitted Huxley's power function in direct scales. Line in log scales. (b) The scatter diagram of residuals against the logarithm of the area. (c) The Q-Q M2N plot of the residuals on a geometric scale. (d, e, and f) Corresponding diagrams for a TAMA-M2N.

Table 8 presents fitting statistics relating to a MEM-LogM2N protocol (cf. Equations (26) and (27)) performing on the EHDS.  Table 9 provides fitting statistics associating with a TAMA-M2N fit (cf. Equations (30) and (31)).  Figure 9(a) presents the spread about the MEM-LogM2N fitted Huxley's form of a systematic part.  Figure 9(b) exhibits the corresponding residual spread.  Figure 9(c) presents the associating Q-Q LogM2N diagram. It becomes evident that a M2N distribution bears a fair model for the residual dispersion. AIC values clearly asses a gradual improvement of quality of fit favoring the M2N-driven protocols, i.e., MEM (AIC = −94745.76), MEM-loglogistic (AIC = −97297.3),  and MEM-LogM2N (AIC = −99520.8). And similarly, for the TAMA related fits (TAMA (AICA = −94745.8) TAMA-logistic (AICA = −97297.2), and TAMA-M2N (AICA = −99521.0)).We may be also aware of improved residual spread relative to the severe heavy tail patterns underlying both the MEM and the MEM-loglogistic schemes.  Figure 9(d) shows the spread about the linear mean response in geometrical space produced by a TAMA-M2N scheme fitted on the EHDS.  Figure 9(e) displays TAMA-M2N residual spread and Figure 9(f) presents the link up with Q-Q M2N plot. The M2N modified TAMA scheme shows a better fit than either the regular TAMA or its logistic-borne counterpart since as we can be ascertained by comparing Q-Q diagrams in Figure 3 through Figure 7, the consistencies of the Q-Q M2N-borne spreads are remarkable, compared to the severe heavy tails patterns displayed by either the Q-Q normal and Q-Q logistic plots.

Besides when comparing the fits with error term produced by the M2N to those driven by the normal or logistic distributions on the arithmetic scale, using the Akaike information criterion (AIC), where the lower value of the AIC index represents a better fit, we can be aware from Tables 1, 3, 4, 6, 8, and 10 that the M2N model presents a better fit than the remaining ones. The above results concede a notable improvement when replacing the normal or logistic distribution forms of the error term in the geometric scale with an M2N counterpart. Yet another advantage of the M2N model concerns to the uncertainty of the estimated parameters, since as we can see in Tables 1–10, the relative standard errors of the estimates of the parameters *α* and *β* of the M2N model are smaller than the ones attaching to the normal or logistic counterparts, that is, the M2N model returns parameter estimates with better precision. Consequently, we can sustain that M2N model surpasses the normal or logistic ones in quality of the fit to the EHDS.

### 3.8. Performance of the Breusch-Pagan and M2N Modification to the Regular DNLR Scheme Fitted on the EHDS

The regression equation associating with a DNLR-BP-M2N scheme becomes the following:

(33) $$\begin{array}{c} y=\beta x^{\alpha }+\epsilon , \end{array}$$

with *ϵ* ~ M2N(*p*, 0, *σ*). For a M2N distribution, the standard deviation becomes the following:

(34) $$\begin{array}{c} \sigma =\sqrt{p\sigma_{1}^{2}+\left(1-p\right)\sigma_{2}^{2}}. \end{array}$$

In order to adapt the model of Equation (19) for the heteroskedastic case, similarly to the Breusch-Pagan [47] test, we modify the standard deviations *σ*_1_ and *σ*_2_, such that one to one take forms *σ*_1_(1 + *kx*) and *σ*_2_(1 + *kx*), to obtain the following:

(35) $$\begin{array}{c} \sigma \left(y\left|x\right.\right)=\sqrt{p{\left[\sigma_{1}+\left(1+kx\right)\right]}^{2}\left(1-p\right){\left[\sigma_{2}\left(1+kx\right)\right]}^{2}} \end{array}$$

or equivalently

(36) $$\begin{array}{c} \sigma \left(y\left|x\right.\right)=\sqrt{p\sigma_{1}^{2}+\left(1-p\right)\sigma_{1}^{2}\left(1+kx\right)} \end{array}$$

The likelihood function of this model can be written as follows:

(37) $$\begin{array}{c} L\left(\beta ,\alpha ,\sigma_{1},\sigma_{2},p,k\right)=\overset{n}{\underset{i=1}{\prod }}\left\{p\frac{1}{\sqrt{2\pi \sigma_{1i}^{2}}}\exp\left[-\frac{1}{2\sigma_{1i}^{2}}{\left(v_{i}-\mu_{i}\right)}^{2}\right]+\left(1-p\right)\frac{1}{\sqrt{2\pi \sigma_{2i}^{2}}}\exp\left[-\frac{1}{2\sigma_{2i}^{2}}{\left(v_{i}-\mu_{i}\right)}^{2}\right]\right\}, \end{array}$$

where *μ*_*i*_ = *βx*_*i*_^*α*^ and *σ*_*ji*_ = *σ*_*j*_(1 + *kx*_*i*_), *j* = 1, 2.

**Figure 10:**
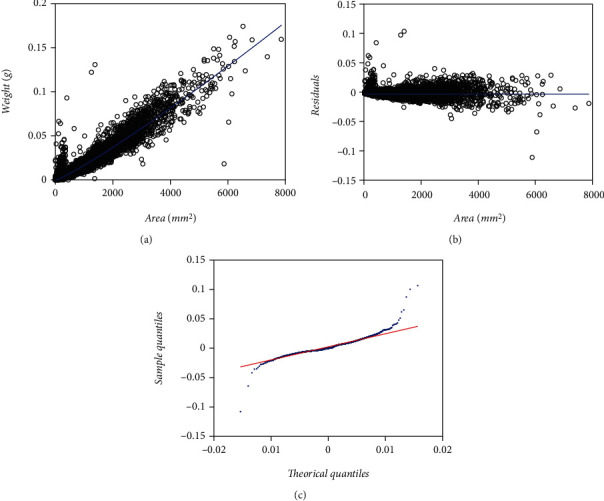
Spread about mean response curve residual and QQ-normal plots of a DNLR-M2N-BP scheme (cf. Equations (33) and (37)) fitted on the Echavarría-Heras et al. [37] data. (a) Dispersion about fitted Huxley's power function form. (b) The scatter diagram of residuals against leaf area. (c) The Q-Q M2N plot of the residuals on direct arithmetical scales. A heavy tails pattern compatible to that appearing in Figure 4(c) prevails.

Table 10 presents the parameter estimates and fitting statistics relating to a DNLR-BP-M2N scheme (cf. Equations (33) and (37)) fitted on the EHDS.  Figure 10 exhibits corresponding spread plots.  Figure 10(a) displays dispersion about fitted Huxley's power function form.  Figure 10(b) shows the scatter diagram of residuals against leaf area.  Figure 10(c) shows Q-Q M2N plot of the residuals on direct arithmetical scales. Spreads about the mean response function and that of residuals around the zero lines seem fair. Compared to the fit of a regular DNLR scheme of Equations (8) and (9), the Q-Q M2N diagram appearing in Figure 10(c) displays improvement regarding the expected pattern for a heavy tailed distribution. But anyhow, an asymmetrical heavy tailed spread persists.  Table 5 explains the improvement of the quality of the fit in terms of the AIC value achieved by the DNLR-M2N fit. In this table, we note that there is a greater reduction when we change from the normal distribution borne DNLR to the DNLR-M2N composite; that is, we find an important change when going from the normal to the M2N distribution, this of course due to the heavy-tailed pattern that exists in the data.

### 3.9. Implementation of the TAMA-Poly(*K*): The *K*th-Degree Polynomial Modification to the Linear Systematic Term of a Regular TAMA Scheme

With the aim of comparing the reproducibility strength of a M2N-driven modification of a TAMA scheme against that entailed by the *K*th-degree polynomial variation to a regular TAMA undertaken by Echavarría-Heras et al. [37]. So, conceived scheme refers through the symbol, TAMA-Poly(*K*), and entails a form of a complex allometry and normally distributed error composite scheme in geometrical space. Formally, a TAMA-Poly(*K*) scheme stands for a modification of the regression model of Equation (2) that establishes through the following:

(38) $$\begin{array}{c} v=\beta_{0}+\overset{K}{\underset{k=1}{\sum }}\alpha_{k}u_{i}^{k}+\epsilon , \end{array}$$

with *ϵ* ~ *N*(0, *σ*), and so, the likelihood function of this model is given as follows:

(39) $$\begin{array}{c} L\left(\beta_{0},\alpha ,\sigma \right)=\overset{n}{\underset{i=1}{\prod }}\frac{1}{\sqrt{2\pi \sigma^{2}}}\exp\left[-\frac{1}{2\sigma^{2}}{\left(v_{i}-\mu_{i}\right)}^{2}\right], \end{array}$$

where *α* = (*α*_1_, ⋯, *α*_*K*_) and *μ*_*i*_ = *β*_0_ + ∑_*k*=1_^*K*^*α*_*k*_*u*_*i*_^*k*^.

This time, the correction factor for retransformation aims becomes the following:

(40) $$\begin{array}{c} \text{CF}=\exp\left(\frac{\sigma^{2}}{2}\right). \end{array}$$

**Figure 11:**
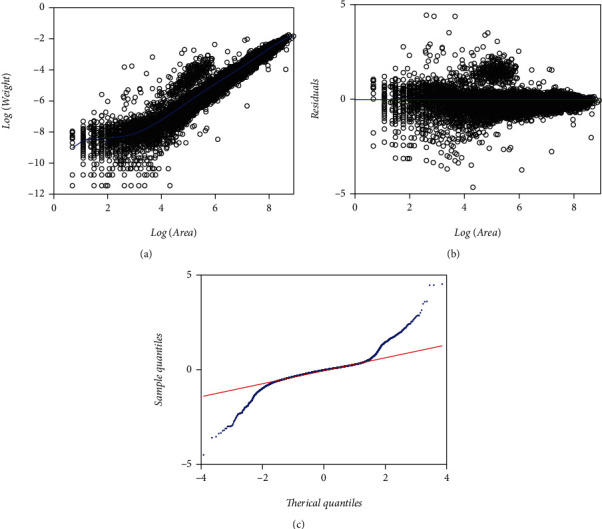
Spreads in geometrical space produced by the TAMA-Poly(6) and the polynomial modified TAMA scheme proposed by Echavarría-Heras et al. [37] when fitted on the EHDS (cf. Equations (38) and (39)). (a) Spread about fitted 6th degree polynomial systematic term, in geometrical space. (b) The scatter diagram of residuals against the logarithm of the area. (c) The associating Q-Q normal plot of the residuals on a geometric scale. Even though fitted systematic part seems to better describe a phase-like heterogeneity, still a heavy tails spread pattern in the Q-Q normal plot shows up.

Table 11 presents fitting statistics of a TAMA-Poly(6) scheme (cf. Equations (38) and (39)) performing on the EHDS.  Figure 11(a) shows the spread about the systematic term, in geometrical space of a TAMA-Poly(6) scheme fitted on the EHDS.  Figure 11(b) shows the scatter diagram of the TAMA- Poly(6) residuals against the logarithm of the area.  Figure 11(c) displays the TAMA-Poly(6) associating Q-Q normal plot of the residuals on a geometric scale. Even though, fitted TAMA-Poly(6) systematic part seems to better describe a phase-like heterogeneity shown in Figure 1(b), still the Q-Q normal plot of Figure 11(c) displays a heavy tails spread pattern. AIC values in Table 8 and Table 10 allow comparison of the TAMA-M2M and the TAMA-Poly(6) models fitted on the EHDS. We can be aware of a difference ΔAIC = 3139 favoring selection of the TAMA-M2M over the TAMA-Poly(6) counterpart. This try conveniently explains the failure of a polynomial-like complex allometry form to normalize the distribution of residuals.

### 3.10. Assessment of Reproducibility Strength of Addressed Methods

We present statistics that allow the assessment of the reproducibility strengths of adapted allometric proxies while projecting values for the assessment of reproducibility strength of allometric proxies for observed monthly average leaf biomass in shots reported in the EHDS (Table 12). We provide AIC index and other model performance metrics, such as the standard error of estimate (SEE) and mean prediction error (MPE) [49–52]. Likewise, we include Lin's concordance correlation coefficient, denoted here through the CCC symbol [53]. Agreement between allometrically projected and measured values will be defined as poor whenever CCC < 0.90, moderate for 0.90 ≤ CCC < 0.95, good for 0.95 ≤ CCC < 0.99, or excellent for CCC ≥ 0.99 [54]. Calculation of reproducibility measures for the TAMA-based methods relied in retransformation of ensuing protocols fitted in geometrical space. We also propose what we call a relative absolute deviation (RAD) index value. To calculate these statistics, we firstly obtain the absolute deviation between the overall mean of monthly averages of observed leaf biomass values and the corresponding one derived allometrically, and then, we divide by the overall mean of monthly averages of observed individual leaf biomass values.

According to Tables 1 and 3 for the normal distribution produced models, it was the MEM one which performed the better. Nevertheless, a MEM row in Table 12 allows assessing the feasibility of assuming that the basic error random variable *ϵ* distributes normally. As we can be aware of, the MEM row implies the less accurate proxies for monthly average leaf biomass in shoots .Certainly among the compared methods, the MEM one arrangement returned the smaller CCC entry and the greater SSE, MPE, MPSE, and RD values. We can also ascertain that enhancing complexity of error structure to contemplate a M2N distribution led to a remarkable reproducibility strength of MEM-LogM2N. Moreover, a difference between a CCC value of the TAMA-Poly(6) slant and the MEM-LogM2N one is of only ΔCCC = 0.0184. Moreover, giving to the SEE, MPE, and RD values the MEM-LogM2N construct shows equivalent reproducibility strength than the TAMA-Poly(6), but the MEM-LogM2N performs slightly better according to the MPSE statistics. Besides, the difference in CCC values between the TAMA-Poly(6) and the DNLR-M2N-BP amounts to only ΔCCC = 0.0069. This is in spite of the fact that the DNLR-BP-M2N composite associates with the largest AIC. Therefore, focusing on the proper error structure determined higher reproducibility features of the M2N-borne protocols.

### 3.11. Simulation Study

In this section, we carry out a study aimed to evaluate the performance of the proposed protocol under a known scenario. For this aim, we adapt a simulation procedure based on the multiplicative allometric model with a mean response of the Huxley power function type and error term according to a logM2N(*p*, 0, *σ*) distribution. The joining formulation is given by Equation (26). The procedure yields pairs including the foliar area values *x*_*i*_ in the EHDS and simulated values of their corresponding leaf biomass replicates *y*_*ij*_, *j* = 1, 2, ⋯, *r*(*i*). To produce the leaf biomass replicates, we use the formula:

(41) $$\begin{array}{c} y_{ij}=\beta {\left(x_{i}\right)}^{\alpha }\exp\left(\epsilon_{ij}\right), \end{array}$$

where *β* and *α* with are the values of the allometric parameters given in Table 8 and for a fixed *x*_*i*_*i* = 1, 2, ⋯, *m* , and for *j* = 1, 2, ⋯, *r*(*i*), *ϵ*_*ij*_ drawn from a M2N(*p*, 0, *σ*) distribution with $\sigma =\sqrt{p\sigma_{1}^{2}+\left(1-p\right)\sigma_{2}^{2}}$ for *p* , *σ*_1_ and *σ*_2_ as listed in Table 8. The EHDS compose *n* = 10450 pairs of measurements of leaf area and related weight. The number of different leaf area values recorded in the sample is *m* = 2295 . Therefore, the vector (*r*(1), *r*(2), ⋯, *r*(*m*)) satisfies ∑_1_^*m*^*r*(*i*) = *n*.

Once we produced the *n* simulated data pairs, we generated samples obtained by randomly removing ten pairs out of the simulated pool. We returned the extracted data so we could deal with the complete collection of simulated data before getting the following sample. The process iterated until the completion of a number of 1000 random samples. For the *i*th sample, we achieved a fit of the model of Equations (26) and (27) to obtain a vector (*β*_*si*_, *α*_*si*_, *p*_*si*_, *σ*_1*si*_, *σ*_1*si*_) of estimated parameters. Then, we acquired the root mean squared errors (RMSE) of so estimated parameters and their reference values (*β*, *α*, *p*, *σ*_1_, *σ*_2_) listed in Table 8. Calculation of RMSE values achieved through the formula:

(42) $$\begin{array}{c} \text{RMSE}\left(\theta_{s}\right)=\sqrt{\left(\frac{1}{n}\right)\overset{n}{\underset{i=1}{\sum }}{\left(\theta_{si}-\theta \right)}^{2}}, \end{array}$$

where *θ* stands for the value of a reference parameter and *θ*_*s*_ for its estimated value through the simulation procedure. Correspondingly *θ*_*si*_  stands for the proxy of *θ*_*s*_ deriving from the associating *i*th sample of data pairs drawn from the simulated pool.

**Figure 12:**
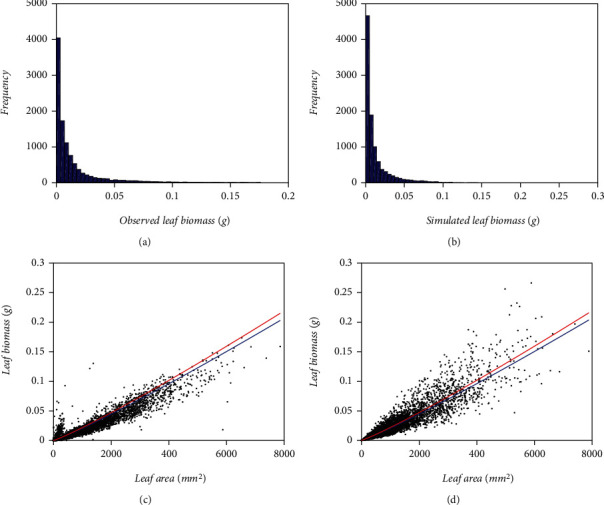
Results of the simulation study. (a, b) Histograms associating with the EHDS and to simulated data one to one. (c) The spread of leaf biomass values relative to the mean response curve obtained by fitting the model of Equations (25) and (26) to the EHDS (red lines) and that produced by fitting the named model to simulated data (blue lines). (d) Equivalent spreads corresponding to simulated data.

We present the statistics resulting from simulation runs in Table 13.  Figure 12 allows visual assessment of reproducibility features of the mean response function fitted from simulated data.  Table 14 allows a comparison of referred reproducibility strength in quantitative grounds. Entries in Table 13 demonstrate that reference values of the allometric parameters in the model of Equations (25) and ((26)**)** can be efficiently approximated through the addressed simulation procedure. Histograms in Figure 2(a) and Figure 2(b) suggest consistency of an M2N distribution for both the EHDS and the simulated data set. We can also be aware from Figure 12(c) and Figure 12(d) that the simulation procedure failed to reproduce the domed-like spread at the beginning of the distribution in the EHDS. This fact readily explains the drop in AIC deriving from simulated data. It also strengthens a perception of a lack of standardization in data gathering while conforming to the EHDS sample. Spreads around the mean response function fitted from simulated data (blue lines) show fairness for both the EHDS and the simulated data. Whatever bias among projections produced by mean response curves explains by the lack of the referred domed-like spread in simulated data. The closeness of reproducibility index values in Table 14 corroborates this judgment. Altogether, the result of addressed simulation runs allows confirming the fairness of the offered M2N-based allometric analysis protocol.

### 3.12. Summary of Results

We extended the approach by Montesinos-López et al. [36] to consider a setup where a mixture of normal distributions drives stochasticity of the error term of the regular TAMA fit. Present data composes (*n* = 10,410) pairs of measurements of eelgrass leaf biomass and corresponding area adapted from a sample originally reported in Echavarria-Heras et al. [37] by removing two data pairs that we considered as unduly outliers (Figure 1). Moreover, at a first glance, we can be aware that present data stands for bigger an extent and variability than contemplated by Montesinos-López et al. [36] (Figure 2).

By looking at a relatively fair spread about the systematic parts of fitted MEM, TAMA, and DNLR-BP schemes (Figure 3(a)), (Figure 4(a)), and (Figure 5(a)), it is reasonably assuming the reliability of Huxley's model in detecting the actual trend in present data. But in spite of this fact, the Q-Q lognormal and Q-Q normal diagrams, respectively, shown in Figure 3(c), Figure 4(c), and Figure 5(c), reveal that a normal distribution assumption for the random variable *ϵ* that determines the error terms to the MEM, TAMA, and DNLR-BP fits (Equations (2), (6), and (10) one to one) does not support a suitable model for the variability pattern inherent to the EHDS. To highlight the relevance of conveying a proper error structure, it is worth stating that assuming an additive error model in direct scales (Equation (10)) did not provide suitability of fit. Indeed, the ensuing DNLR-BP model resulted in a noticeably larger AIC value (AIC = −84528.9) than its MEM equivalent (AIC = −94745.76). Supposedly, the DNLR-BP should entail the same reproducibility features as a MEM counterpart. Nevertheless, when fitted to present data, an inherent multiplicative error structure of the MEM entailed a better performance, independently of the BP adaptation for heteroscedasticity that strengthened the additive DNLR scheme. Moreover, agreeing to Table 15, the DNLR-BP composites registered the most significant absolute values for the Δ*AIC* entries among the different methods.

The logistic distribution can reasonably model a higher dispersion than the normal one [36]. But, heavy tails displayed in the Q-Q loglogistic diagram of Figure 6(c) refer to an unfair MEM-loglogistic fit (Equation (9)). Equally, the Q-Q logistic plot of the residuals of a TAMA-logistic fit (Figure 6(f)) on geometrical space showing heavy tails also points to an inconsistent TAMA-logistic (Equation (13)) fit. Comparing AIC values among the MEM-logistic (AIC = −97297.3) and the DNLR-BP-logistic (AIC = −94549) favors the MEM-logistic (Table 15). Therefore, even switching to a logistic distribution, a perception of the unsuitability of an additive error structure maintains. Certainly, Figure 7(c) displays a heavy-tailed pattern, asymmetrical as in a DNLR-BP fit (cf. Figure 5(c)). Again, such an asymmetrical spread Figure 7(c) for the DNLR-BP-logistic scheme could explain by the attempt to model the actual heteroscedastic spread of Figure 1 through a BP-logistic adapted DNLR additive error model. And on top of that, Lai et al. [40] observation of biased direct nonlinear regression for large covariate values could also be pertinent. In any event, the spreads in the Q-Q loglogistic in Figure 6(c) and QQ-logistic plots in Figure 6(f) and Figure 7(c) bear that assuming a logistical distribution-driven error term also turns out to be unfeasible.

The MAD technique adapted by Echavarria-Heras et al. [28] delivered a quality-controlled data set missing about 25% of the total replicates in the crude EHDS. But, even though the MAD procedure removed a large share of the original data, the spread in Figure 8(a) and Figure 8(b) suggests the unreliability of a TAMA protocol when fitted to processed data. Still, regardless of an apparent curvature indicated by these spread plots, Figure 8(c) depicts a symmetric heavy tails arrangement. Therefore, that unravels that a TAMA fit failed to normalize the residual dispersion on processed data. Consequently, due to this fact and being aware of the fitting results of the protocols addressed so far, a conclusion seems indubitable. If the aim is to explore the possibility of maintaining Huxley's model-like systematic term in the analysis of the EHDS, we definitely ought to adapt the error driving random variable *ϵ* to conform to a symmetric distribution with zero mean and heavy tails. This perspective endorses consideration of a mixture of two normal distributions with zero mean and different deviations as a reasonable model for the randomness of *ϵ*.

Table 15 allows comparison of the fits based on an M2N distribution to those related to the normal or logistic ones on the arithmetic scales, using the Akaike information criterion, which entails the M2N-shouldered models as better suited than the remaining ones. For instance, for composites involving a MEM, the reliability of fits remarkably improved when we switched from normal to logistic and then to a M2N model for the random variable *ϵ*, (i.e., MEM (AIC = −94745.76), MEM-loglogistic (AIC = −97297.3), and MEM-LogM2N (AIC = −99520.8)). Similarly, Tables 2, 5, 9, and 11 display the AIC statistics associated with fits in geometrical scales, showing that the TAMA-M2N entailed the best fit even when compared to the TAMA-Poly(6) arrangement (i.e., TAMA (AIC = 17702.4), TAMA-Poly(6) (AIC = 16066.5), TAMA-logistic (AIC = 15150.9), and TAMA-M2N (AIC = 12927.2)). Likewise, Tables 3, 6, and 10 show that for the DNLR-borne protocols, the quality of fits also improved when moving from normal to logistic and then to a M2N distribution (DNLR-BP (AIC = −84528.9), DNLR-BP-logistic (AIC = −94549), and DNLR-BP-M2N (AIC = −93309.8)). Moreover, the QQ-LogM2N plot shown in Figure 9(c) resulting from a fit of the MEM-LogM2N scheme (Equations (26) and (27)) already displays a fair spread. Similarly, Figure 9(f) showing the Q-Q M2N diagram corresponding to a TAMA-M2N fit (Equations (30) and (31)) indicates a spread unswerving to assumed M2N distribution. Tables 1–6 and 8–10 reveal that the relative standard errors of the estimates of the parameters *α* and *β* of the M2N model are smaller than those added to the normal or logistic counterparts. Moreover, reproducibility index values listed in Table 12 explain the advantage of the M2N distribution for a driving of good reproducibility strength of projections of averages of leaf biomass. This distribution enhanced the suitability of the DNLR-based methods even though the error structure implied by this protocol turned on unsuited. Indeed entries in Table 12 confirm that the DNLR-M2N-BP construct beats the TAMA-Poly(6) alternate in SEE, MPE, MPSE, and RD statistics. Additionally, comparison of CCC values in Table 12 unravels that the reproducibility power of a DNLR-BP-M2N rates equivalent to the one calculated for a TAMA-Poly(6) arrangement. Therefore, the M2N model outstrips its normal or logistic partners in the quality of the fit on the EHDS.

The statistics in Table 13 demonstrate that fitted reference values of the allometric parameters in the model of Equations (25) and (26) can be efficiently approximated through simulation procedures. Whatever bias among projections produced by mean response curves portrayed in Figure 12(b) and Figure 12(c) explains since the simulation procedure failed to reproduce a domed-like spread for the smaller leaf biomass values shown in the EHDS. In any event, differences in spreads among the EHDS and its simulated pool could provide further support to our judgment of a lack of systematization while gathering the former sample. Nevertheless, the closeness of reproducibility index values in Table 14 sustains a judgment that bias appointed by named differences in spreads bears irrelevant for practical purposes such as the allometric projection of leaf biomass values. Altogether, the result of addressed simulation runs allows confirming the fairness of the offered M2N-based allometric analysis protocol.

This study confirms a view by Montesinos-López et al. [36] that clinging to the appropriate error structure can offer the reliability of Huxley's model given a particular data set. Indeed, as we demonstrated here, it is possible to enhance the goodness-of-fit in allometric examination by holding up to Huxley's original theoretical perspective, that is, without recurring to the customary complex allometry and normal distribution approaches. It is also worth emphasizing that the presently offered analytical approach allows keeping the parsimonious structure of Huxley's model, thereby facilitating the exploration of a time invariance of the ensuing allometric relationship between eelgrass leaf biomass and area. This feature is paramount to genuinely nondestructive assessments of leaf biomass being relevant to eelgrass conservation.

## 4. Discussion

Huxley's power function model (cf. Equation (1)) beholds great relevance in biology. It offers a theoretical basis for static allometry that is conceived as the assessment of scaling relationships among individuals of a species, e.g., between one organ and total body size [55–57]). Huxley's model also provides a formal scheme for studies on evolutionary allometry that addresses scaling relationships between the sizes of organs of individuals across species [58–60]. And from a purely empirical perspective, whenever a time invariance of the parameters *α* and *β* upholds, Huxley's model can produce convenient nondestructive surrogates of the values of a given allometric response *y*  by using previously fitted estimates and currently taken measurements of a descriptor variable *x* [61–63].

One relevant research subject circumscribing Huxley's model concerns the interpretation of the parameters *α* and *β*. For instance, Huxley stated that the intercept *lnβ* of the line deriving by log transformation on both sides of Equation (1) was of no particular importance. Still, the slope *α* was sufficient to mean static allometry on its own [2, 64]. Furthermore, Huxley's perspective prevails in ongoing research as the valid theoretical standpoint for static allometry [59, 60, 65]. Another vital research subject around Huxley's model concerns the accuracy of estimates of the parameters *α* and *β*, which also relates to the suitability of the analytical scheme to get them. Supporters of the traditional perspective tie to a notion that an allometric response expressed through Equation (1) adapts to a multiplicative growth pattern in the direct scales of data (cf. Equation (2)) which also backs the TAMA (cf. Equation (6)) as the ordinary way to acquire estimates of *α* and *β* [40, 43, 60, 66–73]. But, other opinions sustain that a TAMA approach produces biased results, claiming that since the power function fostering Huxley's model is intrinsically nonlinear, the analysis should instead rely on a DNLR scheme (cf. Equation (8)) [74–77]. Moreover, there are settings where Huxley's model fails to produce a consistent fit. Exploring the reasons undermining the predictive power of this paradigm also endures a prime research subject. One attempt to address a lack of fit in Huxley's model adopts a standing that steers away from covariation among different traits, conceiving allometry as aligned on the covariation between size and shape [78, 79]. From this standpoint, the analysis must rely upon regression schemes that include a systematic term expressed through modifications of Huxley's power function. So conceived variants engender constructs recognized as multiple parameter complex allometry forms (e.g., [75, 80–84]). Yet, since power series offer convenient representations of analytic functions, it turns reasonable to extend the complexity of Huxley's model to adapt polynomial regression schemes in geometrical space [37, 71, 85–88]. But, embracing complex allometry attempts nurtures one of the most irreconcilable disagreements among schools of allometric examination. Indeed, for supporters of a TAMA way, examinations based on complex allometry constructs identified in the direct scales of data lose interpretation of biological theory to honor statistical appropriateness [59, 60, 73]. Besides defenders of traditional allometry claim that Huxley himself offered an approach to extending complexity while maintaining the original theoretical essence of static allometry. Indeed, when exploring the spread in the log-log plot of chela mass vs. body mass of fiddler crabs (Uca pugnax), Huxley acknowledged a breakpoint that was explained by an abrupt change in relative growth of the chela and assumed to take place around the time crabs reached sexual maturity [1, 2, 89]. The idea of a biphasic breakpoint-determined biological scaling conveyed the notion of non-log-linear allometry [90–95]. Extension of Huxley's original idea of a biphasic scaling led to considering multiple breakpoints which in turn spawned the notion of polyphasic log-linear allometry [96–102]. Broken-line regression techniques [37, 103–108] could deliver identification of breakpoints in polyphasic log-linear allometry schemes. Nevertheless, by relying upon nonlinear regression, broken-line schemes require starting values for the break-point estimation, so local maxima and inferences on estimates could make implementation difficult [107, 109]. Then, even though polyphasic log-linear allometry adapts complexity by composing sub-models, each one bearing Huxley's original theoretical envisioning, parameter estimation difficulties could make this approach problematic. Likewise, addressing complex multiparametric allometric forms could return overfitting, i.e., the statistical error of overestimation of the covariate's influence on determining the variability of the allometric response. So, in the tradeoffs to gaining reproducibility strength, overfitting could only offer an approach that overemphasizes empirical relevance, useful in reference only to the current data but not necessarily `to any other data sets. The present results illustrate an approach that enhances the reproducibility strength of Huxley's model while getting around overfitting associating with multiparameter complex allometry forms.

Maintaining Huxley's model within allometric examination offers relevant advantages for eelgrass conservation. Hamburg and Homann [110] used an allometric relationship to express leaf dry weight in eelgrass as a function of length and corresponding width. Solana-Arellano et al. [111] demonstrated that such a two-dimensional allometric dependence derives by assuming the validity of Huxley's laws of proportional growth between the leaf dry weight and the corresponding length and width one by one. Moreover, the leaf architecture in eelgrass approximates the related area as the product of the length times the associated width. Then, it is possible to call in Huxley's original setup and express eelgrass leaf dry weight as an allometric response of the linked area [112]. Likewise, the belt-shaped leaf expansion in eelgrass makes the corresponding length a good allometric descriptor of the dry weight [36]. Substantiation of these approaches allows the adaptation of allometric methods for the nondestructive estimation of aerial eelgrass biomass. These assessments are essential in remediation endeavors given current anthropogenic influences that threaten eelgrass permanence. But, given the general outline above, eelgrass allometric assessment methods based on Huxley's model are the subject of influences that affect their accuracy, precision, and pertinence. Remarkably, a quest for improving fit suitability of the sustaining Huxley's power function may suggest addressing multiparametric complex allometry forms pertinent, thereby drifting away from the theoretical perspective of Huxley's model of simple allometry [37]. It is worth emphasizing that focusing on Huxley's power function embraces parsimony that facilitates a concise exploration of an invariability property of the allometric parameters *α* and *β*, a condition upon which nondestructive assessments of eelgrass leaf biomass hinge in an essential manner.

Furthermore, the spread of present eelgrass leaf biomass to area data suggests strengthening the view by Montesinos-López et al. [36] of focusing on the suitability of error distribution to amend a lack of fit of Huxley's model. Indeed, it is worth recalling that the present data (EHDS) conforms to an extensive sample of (*n* = 10410) pairs of similar measurements to Montesinos-López et al. [36] data (MLDS) (*n* = 537), both collected at the Punta Banda estuary. They were, moreover, sampling to acquire the EHDS spanning over a complete year cycle, while MLDS stands for only one month. Therefore, as Figure 1(a) portrays, the Echavarria-Heras et al. [37] data set conforms to a remarkably more significant variability than that corresponding to the Montesinos-López et al. [36] sample (Figure 2(a)). Moreover, considerable variability in the EHDS could explain a lack of fit of a TAMA try reported by Echavarria-Heras et al. [37] and which we corroborated here. According to what these authors pointed out, our analysis of the EHDS also lets us become aware of apparent curvature in geometrical space. Likewise, we observed heterogeneity among dispersion patterns associated with the pools of smaller and larger leaves in the sample by analyzing the residual plot. Therefore, agreeing to a customary slant, in researching the EHDS, looking for complex allometry and a normal distribution approach seemed reasonable. But instead of attempting to amend TAMA's inconsistency based on a broken-line protocol or, more specifically, through a polynomial regression scheme as suggested in Echavarria-Heras et al. [37], we considered it adequate to keep Huxley's form of the systematic component while embedding the error term to nonnormal distribution, just as Montesinos-López et al. [36] devised when advocating for a logistic distribution-borne error term. But, for present data, residual dispersion accompanying a logistically distributed error term in geometrical scales could not improve a heavy tails pattern conforming to the normality of residues. We have to stress two points because the logistic error adaptation of TAMA by Montesinos-López et al. [36] failed to fit the present data well. First of all, as we have already pointed out, the EHDS is considerably larger in sampling dates, number of replicates, and number of processing participants than the MLDS. As a result, we stress that the present analysis deals with data expressing a significantly greater variability than that Montesinos-López et al. [36] dealt with. Second, heterogeneity of spread patterns among smaller and larger leaf sizes is more pronounced in the EHDS than it is in the MLDS. Perhaps this fact could explain the unsuitability of a unimodal distribution as a model for the random variable *ϵ* shaping the error term in present examination. Accordingly, at analyzing present data, a mixture of two normal distributions having a common zero mean but different standard deviations turned on suitable. The present review demonstrates that resultant allometric proxies of monthly averages of eelgrass leaf biomass exhibited similar reproducibility strength to those derived from the TAMA-Poly(6) scheme endorsed by Echavarría-Heras et al. [37].

Echavarría-Heras et al. [37] also advocated the advantages of a median absolute deviation-based data cleaning procedure to remove inconsistent replicates in the present data set. Indeed, we have stressed the significant heterogeneity in the variability of present leaf biomass replicates above. Perhaps such a spread ties to the participation of multiple data processing agents. Compared to Figure 1(a), the spread plots once data cleaning presented in Echavarría-Heras et al. [37] and Figure 8 here undoubtedly display a heteroscedastic pattern consistent with Huxley's model of simple allometry (cf. Equation (2)). Nevertheless, adjusting to a coherent spread to such a model was achieved only upon removing about 25% of the total number of replicates. Deletion of apparently undue replicates seems excessive, thereby questioning the pertinence of data cleaning at all as a suitable model improvement device. Then in analyzing the EHDS, data processing deems dubious even though Huxley's power function stands for the sound biological paradigm, masked by an overwhelming variability of replicates. It is worth adding that the markedly different spreads among smaller and larger leaf sizes in Figure 2(b) for the MLDS could have made it reasonable to call in data cleaning procedures. Nevertheless, as Montesinos-López et al. [36] demonstrated, a fit of a TAMA-logistic scheme in crude data turned on reliable. Furthermore, our results show that a revision of the complexity of error structure was enough to unmask the actual Huxley's power function-driven systematic trend despite a noticeable variability in crude data. Indeed, adapting the complexity of the error term distribution proposed here makes it unnecessary to call in data processing approaches to amend the reproducibility strength of a failing TAMA try. Then present results strengthen a perception of the appropriateness of the procedure suggested by Montesinos-López et al. [36]. Thus, it is possible to amend a lack of fit of a TAMA attempt without recurring to a non-log-linear complex allometry setup.

## 5. Conclusions

We have demonstrated that adapting the complexity of error structure could get around relying on multiple-parameter complex allometry forms as a mechanism to amend a lack of fit of Huxley's model. Maintaining this paradigm at the core of regression schemes aiming to identify scaling relationships offers a way to avoid the overfitting that could associate with using complex allometry-based amendments. For instance, inconveniencies could impair the corroboration of the time invariance of the parameters of the allometric relationship that substantiates nondestructive eelgrass aerial biomass assessments. Even though a lack of normality of residuals in the allometric examination is profusely acknowledged (e.g., [92, 113–116]), it is perhaps a drive to take advantage of the well-established assortment of statistical methods based on the normal distribution that mainly explains refraining from developing analytical tools conforming to alternative residual dispersion models. Given the present findings, it is pertinent to emphasize the advantages of the approach suggested by Montesinos-López et al. [36]. Certainly, allometric proxies for eelgrass leaf biomass produced by Huxley's model and normal mixture distribution composite delivered a reproducibility strength derived from complex allometry and normal distribution alternates [37]. But, intending to achieve empirical pertinence while keeping the parsimony entailed by Huxley's model, the present approach relies upon an error distribution assumption that requires elaboration from a theoretical perspective of allometry itself. Indeed, the problem of selecting the suitable regression model in allometry entails a statement about error structure. This problem remains unaddressed because, in allometry, error structure essentially depends on the inherent biological model and could not be resolved from statistical criteria alone [17–118]. Perhaps the differences in spread patterns among smaller and larger leaf sizes that display in Figure 1(b) and Figure 2(b) could be the entrance to the path to providing a meaning to the involvement of a normal mixture distribution. To get substance to such an adaptation, we should rely on Huxley's quotation of breakpoint allometry itself. Indeed, the spread of present data in geometrical scales and corresponding to the MLDS suggests such a pattern. Indeed, it is reasonable to extend Huxley's original breakpoint idea so that we can justify the envisioning of a similar complexity of an error structure. Concisely for present data, we could assume that each of the pools of leaf sizes splitting by an identified breakpoint fits a parametrized normal distribution differently. The spreads of the collections before and after the breakpoint identified in the present data [37] corroborate suggested heterogeneity. Nevertheless, such a justification could spell a too simplistic rationale for embedding the mixture of two normal distributions in the allometric realm. But given the advantages that the present approach entails in identifying and validating traditional static allometry schemes for eelgrass conservation, it is worth exploring further substantiation and also the possibility of expanding its applicability elsewhere. Therefore, addressing further research on the subject is encouraged, and we particularly plan to undertake these tasks in upcoming contributions.

## Figures and Tables

**Table 1:** Estimated parameters, related statistics, and AIC values associated with a MEM scheme as given by Equations (2) and (3) and fitted on the EHDS. We include parameter estimates with their projected uncertainties, *t* value, *p* value, LogLikMx, and AIC stand for maximum loglikelihood and Akaike information index values one to one.

Parameter	Estimate	Std. err.	Confidence interval (95%)	*t* value	*p* value
*β*	1.3543*e* − 05	2.8836*e* − 07	(1.2978*e* − 05, 1.4108*e* − 05)	47.0	<1.0 × 10^−30^
*α*	1.0239*e* + 00	3.6235*e* − 03	(1.0168*e* + 00, 1.0310*e* + 00)	282.5	<1.0 × 10^−30^
*σ*	5.6609*e* − 01	3.9231*e* − 03	(5.5840*e* − 01, 5.7378*e* − 01)	144.3	<1.0 × 10^−30^
LogLikMx	47375.9				
AIC	-94745.76				

**Table 2:** Estimated parameters and related statistics associating with a TAMA scheme as given by Equations (6) and (7) and fitted on the EHDS. We include parameter estimates with their projected uncertainties, *t* value, *p* value, LogLikMx, and AIC stand for maximum loglikelihood and Akaike information index values one to one. LogLikMxA and AICA correspond one to one to values of the LogLikMx and AIC statistics expressed in direct arithmetical scales.

Parameter	Estimate	Std. err.	Confidence interval (95%)	*t* value	*p* value
*β*_0_	-11.2096	0.021515	(-11.251357, -11.167892)	-520.66	<1.0 × 10^−30^
*α*	1.0239	0.0036615	(1.016808, 1.031013)	279.33	<1.0 × 10^−30^
*σ*	5.6609*e* − 01	0.0039234	(0.558420, 0.573800)	144.3	<1.0 × 10^−30^
LogLikMx	-8848.2				
AIC	17702.4				
LogLikMxA	47375.9				
AICA	-94745.8				

**Table 3:** Estimated parameters, related statistics, and AIC values associating with a DNLR-BP scheme (cf. Equations (10) and (12)) fitted on the EHDS. We include parameter estimates with their estimated uncertainties, *t* value, *p* value, LogLikMx, and AIC stand for maximum loglikelihood and Akaike information index values one to one.

Parameter	Estimate	Std. err.	Confidence interval (95%)	*t* value	*p* value
*β*	9.76*e* − 06	3.76*e* − 07	(9.048*e* − 06, 1.052*e* − 05)	26.0	<2 × 10^−16^
*α*	1.0883	0.0053	(1.078, 1.0987)	205.73	<2 × 10^−16^
*σ*	0.002557	3.4042*e* − 05	(0.00249, 0.00262)	75.2	<2 × 10^−16^
*k*	0.001215	4.1431*e* − 05	(0.001134, 0.001296)	29.4	<2 × 10^−16^
LogLikMx	42268.43				
AIC	-84528.9				

**Table 4:** Estimated parameters, related statistics, and AIC values associating with a MEM-loglogistic scheme (cf. Equations (13) and (14)) fitted on the EHDS. We include parameter estimates with their estimated uncertainties, *t* value, *p* value, LogLikMx, and AIC stand for maximum loglikelihood and Akaike information index values one to one.

Parameter	Estimate	Std. err.	Confidence interval (95%)	*t* value	*p* value
*β*	1.0869*e* − 05	2.1875*e* − 07	(1.0440*e* − 05, 1.1298*e* − 05)	49.7	<1.0 × 10^−30^
*α*	1.0584*e* + 00	3.2813*e* − 03	(1.0520*e* + 00, 1.0648*e* + 00)	322.6	<1.0 × 10^−30^
*σ*	2.6506*e* − 01	2.2582*e* − 03	(2.6063*e* − 01, 2.6949*e* − 01)	117.7	<1.0 × 10^−30^
LogLikMx	48651.65				
AIC	-97297.3				

**Table 5:** Estimated parameters, related statistics, and AIC values associating with a TAMA-logistic scheme (cf. Equations (17) and (18)) fitted on the EHDS. We include parameter estimates with their estimated uncertainties, *t* value, *p* value, LogLikMx, and AIC stand for maximum loglikelihood and Akaike information index values one to one. LogLikMxA and AICA correspond one to one to values of the LogLikMx and AIC statistics expressed in direct arithmetical scales.

Parameter	Estimate	Std. err.	Confidence interval (95%)	*t* value	*p* value
*β*_0_	-11.4296	0.02007617	(−1.1469*e* + 01, −1.1390*e* + 01)	-569.3	<1.0 × 10^−30^
*α*	1.0584*e* + 00	3.2813*e* − 03	(1.0520*e* + 00, 1.0648*e* + 00)	322.6	<1.0 × 10^−30^
*σ*	2.6506*e* − 01	2.2582*e* − 03	(2.6063*e* − 01, 2.6949*e* − 01)	117.7	<1.0 × 10^−30^
LogLikMx	-7572.45				
AIC	15150.9				
LogLikMxA	48651.6				
AICA	-97297.2				

**Table 6:** Estimated parameters, related statistics, and AIC values associating with a DNLR-BP-logistic scheme of Equations (20) and (22) fitted on the EHDS. We include parameter estimates with their estimated uncertainties, *t* value, *p* value, LogLikMx, and AIC stand for maximum loglikelihood and Akaike information index values one to one.

Parameter	Estimate	Std. err.	Confidence interval (95%)	*t* value	*p* value
*β*	7.077*e* − 06	1.702*e* − 07	(6.744*e* − 06, 7.411*e* − 06)	41.59	<2 × 10^−16^
*α*	1.129*e* + 00	3.538*e* − 03	(1.122*e* + 00, 1.136*e* + 00)	319.16	<2 × 10^−16^
*σ*	2.702*e* − 04	9.855*e* − 06	(2.508*e* − 04, 2.895*e* − 04)	27.41	<2 × 10^−16^
*k*	9.651*e* − 03	4.879*e* − 04	(8.694*e* − 03, 1.061*e* − 02)	19.78	<2 × 10^−16^
LogLikMx	47279				
AIC	-94549				

**Table 7:** Estimated parameters and related statistics associating with a TAMA scheme as given by Equations (6) and (7) and fitted on the processed EHDS. We include parameter estimates with their estimated uncertainties, *t* value, *p* value, LogLikMx, and AIC stand for maximum loglikelihood and Akaike information index values one to one. LogLikMxA and AICA correspond one to one to values of the LogLikMx and AIC statistics expressed in direct arithmetical scales. Statistics on the processed data are only provided for completion of the presentation and do not intend to support comparison to the remaining fits as those relied upon the crude EHDS.

Parameter	Estimate	Std. err.	Confidence interval (95%)	*t* value	*p* value
*β*_0_	-11.3663610	0.01669583	(-11.39908, -11.33364)	-680.79	1.0 × 10^−30^
*α*	1.044361	0.00278709	(1.03890, 1.04982)	374.71	1.0 × 10^−30^
*σ*	0.382246	0.00305903	(0.37625, 0.38824)	124.96	1.0 × 10^−30^
LogLikMx	-3569.7				
AIC	7145.4				
LogLikMxA					
AICA					

**Table 8:** Estimated parameters, related statistics, and AIC value associating with a MEM-LogM2N scheme (cf. Equations (26) and (27)) fitted on the EHDS We include parameter estimates with their estimated uncertainties, *t* value, *p* value, LogLikMx, and AIC stand for maximum loglikelihood and Akaike information index values one to one.

Parameter	Estimate	Std. err.	Confidence interval (95%)	*t* value	*p* value
*β*	7.7446*e* − 06	1.4350*e* − 07	(7.4633*e* − 06, 8.0259*e* − 06)	54.0	<1.0 × 10^−30^
*α*	1.1106*e* + 00	2.9287*e* − 03	(1.1049*e* + 00, 1.1163*e* + 00)	379.2	<1.0 × 10^−30^
*p*	2.1182*e* − 01	7.0370*e* − 03	(1.9803*e* − 01, 2.2561*e* − 01)	30.1	<1.0 × 10^−30^
*σ*_1_	1.1682*e* + 00	2.2715*e* − 02	(1.1237*e* + 00, 1.2127*e* + 00)	51.4	<1.0 × 10^−30^
*σ*_2_	2.6053*e* − 01	3.0627*e* − 03	(2.5453*e* − 01, 2.6653*e* − 01)	85.1	<1.0 × 10^−30^
LogLikMx	49765.43				
AIC	-99520.85				

**Table 9:** Estimated parameters, related statistics, and AIC value associating with a TAMA-M2N protocol (cf. Equations (30) and (31)) fitted on the EHDS. We include parameter estimates with their estimated uncertainties, *t* value, *p* value, LogLikMx, and AIC stand for maximum loglikelihood and Akaike information index values one to one. LogLikMxA and AICA correspond one to one to values of the LogLikMx and AIC statistics expressed in direct arithmetical scales.

Parameter	Estimate	Std. err.	CI (95%)	*t* value	*p* value
*β*_0_	-11.7704676	0.0185185	(-11.806764, -11.734171)	-635.6	< 5 × 10^−30^
*α*	1.1108684	0.0029282	(1.105129, 1.116608)	379.4	< 5 × 10^−30^
*p*	0.7914487	0.0069130	(0.777899, 0.804998)	114.5	< 5 × 10^−30^
*σ*_1_	0.2614560	0.0030538	(0.255471, 0.267442)	85.6	< 5 × 10^−30^
*σ*_2_	1.1918691	0.0232128	(1.146372, 1.237366)	51.3	< 5 × 10^−30^
LogLikMx	-6458.6				
AIC	12927.2				
LogLikMxA	49765.5				
AICA	-99521.0				

**Table 10:** Parameter estimates of the DNLR-BP-M2N and the heteroscedastic [47] nonlinear model with the additive error term distributed as the presently addressed mixture of two normal distributions (cf. Equations (33) and (37)). We include parameter estimates with their estimated uncertainties, *t* value, *p* value, LogLikMx, and AIC stand for maximum loglikelihood and Akaike information index values one to one.

Parameter	Estimate	Std. err.	CI (95%)	*t* value	*p* value
ln*β*	−1.1969494*e* + 01	1.7554895*e* − 02	(−1.2003901*e* + 01, −1.1935087*e* + 01)	-681.8	2 × 10^−16^
*β*	6.3345341*e* − 06	1.1120208*e* − 07	6.1165821*e* − 06, 6.5524862*e* − 06	56.9	2 × 10^−16^
*α*	1.1441543*e* + 00	2.6137715*e* − 03	1.1390314*e* + 00, 1.1492772*e* + 00	437.7	2 × 10^−16^
*p*	0.77420	0.006906	(0.76067, 0.78774)	112.2	2 × 10^−16^
*σ*_1_	2.8736203*e* − 03	1.1726859*e* − 04	2.6437781*e* − 03, 3.1034625*e* − 03	24.5	2 × 10^−16^
*σ*_2_	1.6145331*e* − 04	4.2346650*e* − 06	1.5315352*e* − 04, 1.6975310*e* − 04	38.1	2 × 10^−16^
*k*	2.1505795*e* − 02	6.6892421*e* − 04	2.0194728*e* − 02, 2.2816862*e* − 02	32.1	2 × 10^−16^
LogLikMx	50992.90				
AIC	-101973.81				

**Table 11:** Fitting results of a TAMA-Poly(6) regression protocol based on a 6th degree polynomial systematic term modification to the regular TAMA scheme (cf. Equations (38) and (39)) performing on the Echavarría-Heras et al. [37] data set. We include parameter estimates with their estimated uncertainties, *t* value, *p* value, LogLikMax, and AIC stand for maximum loglikelihood and Akaike information index values one to one. LogLikMxA and AICA correspond one to one to values of the LogLikMx and AIC statistics expressed in direct arithmetical scales.

Parameter	Estimate	Std. err.	Confidence interval (95%)	*t* value	*p* value
*β*_0_	-11.748088	0.463156	(−1.26559*e* + 01, −1.084030*e* + 01)	-25.365	<1.0 × 10^−30^
*α*_1_	6.112240	0.818839	(4.507316, 7.717164)	7.4645	<1.0 × 10^−13^
*α*_2_	-4.426333	0.547929	(-5.500274, -3.352392)	-8.07829	<1.0 × 10^−15^
*α*_3_	1.516275	0.179640	(1.164181, 1.868369)	8.4406	<1.0 × 10^−16^
*α*_4_	-0.252736	0.030866	(-0.3132334, -0.1922386)	-8.18816	<1.0 × 10^−15^
*α*_5_	0.020574	0.0026667	(0.015300, 0.025800	7.715	<3.0 × 10^−8^
*α*_6_	-0.0006561	9.13681*e* − 05	(−8.35182*e* − 04, −4.77019*e* − 04)	-7.18084	<7.0 × 10^−13^
*σ*	0.523300	0.00362759	(0.516100, 0.5303)	144.2431	<1.0 × 10^−30^
LogLikMx	-8025.2				
AIC	16066.5				
LogLikMxA	48198.9				
AICA	-96381.8				

**Table 12:** Statistics for the assessment of the reproducibility strengths of the presently acquired allometric proxies while projecting monthly averages of observed eelgrass leaf biomass in shoots as reported in the EHDS. We include Akaike information criterion (AIC) index, Lin's concordance correlation coefficient (CCC), standard error of estimate (SEE), mean prediction error (MPE), and relative deviation (RD). Comparison restricts to better performing proxies identified in arithmetical scales as well as the retransformed TAMA-Poly(6) scheme.

Model	Table	AIC	CCC	SEE	MPE	MPSE	RD
MEM	1	-94745.76	0.9174	0.0039	15.02	16.74	0.1410
MEM-loglogistic	4	-97297.3	0.9432	0.0033	12.65	13.94	0.1158
DNLR-BP-logistic	6	-84528.9	0.9864	0.0016	6.48	7.96	0.0015
MEM-LogM2N	7	-99520.8	0.9712	0.0024	9.2443	10.53	0.0801
DNLR-BP-M2N	9	-93309.8	0.9827	0.0019	7.28	8.67	0.0550
TAMA-Poly(6)	10	-96381.8	0.9896	0.0023	8.9115	10.74	0.0826

**Table 13:** Values of the reference parameters *β*, *α*, *p* , *σ*_1_, and *σ*_2_ obtained by fitting the model of Equations (25) and (26) to the EHDS (EHDS row) and the corresponding ones; *β*_*s*_, *α*_*s*_, *p*_*s*_, *σ*_1*s*_, and *σ*_1*s*_ deriving from simulated data (simulated row). We include RMSE values calculated through Equation (42) (RMSE row) and the RMSE relative to reference parameter values (relative RMSE row).

Parameter	*α*	*β*	*σ*_1_	*σ*_2_	*p*
EHDS	7.7446*e* − 06	1.1106*e* + 00	1.1682*e* + 00	2.6053*e* − 01	2.1182*e* − 01
Simulated	9.5647*e* − 06	1.1111*e* + 00	3.2352*e* − 01	3.2352*e* − 01	2.0045*e* − 01
RMSE	3.5543*e* − 09	6.3267*e* − 05	6.8730*e* − 05	6.9691*e* − 05	9.0712*e* − 03
Relative RMSE	0.03716098	0.00569383	0.02124381	0.02154086	4.53564111

**Table 14:** Reproducibility strength of allometric proxies of eelgrass leaf biomass values in the EHDS produced by the model of Equations (25) and (26) fitted to real data (EHDS row) compared to corresponding values arising from named model fitted to simulated data (simulated data row). We include Akaike information criterion (AIC) index, Lin's concordance correlation coefficient (CCC), standard error of estimate (SEE), and mean prediction error (MPE).

Agreement index	AIC	CCC	SEE	MPE
EHDS	-99520.8	0.9495	0.0065	0.9940
Simulated data	-103320.71	0.9382	0.0075	1.1383

**Table 15:** Akaike information criterion values for comparison of the different protocols in direct scales contemplated in the present examination. AIC values appear in the intersection of rows for models and columns for the distribution of fundamental error random variable *ϵ*. For the TAMA, TAMA-logistic, TAMA-M2N, and TAMA-Poly(6) models fitted in geometrical space, we use the corresponding AICA values listed in Table 2, Table 5, Table 9, and Table 11 one to one. The ΔAIC entries to each AIC represent the difference in the AIC of each method relative to the smallest AIC value, that is, the one associated with the TAMA-M2N composite.

Model-Dist. (*ϵ*)	Normal	ΔAIC	Logistic	ΔAIC	M2N	ΔAIC
MEM	-94745.76	-4775	-97297.3	-2301	-99520.8	0
TAMA	-94745.8	-4775	-97297.2	-2301	-99521.0	-0.2
DNLR-BP	-84528.9	-14992	-94549	-4972	-93309.8	-6211.2
TAMA-Poly(6)	-96381.8	-3410				

## Data Availability

Data will be available from the corresponding author upon request.

## Appendix

### A. Model Performance Metrics

In addition to the AIC and CCC statistics, model assessment here takes into account on the SEE, MPE, and MPSE indices that rely on statistics of squared and absolute deviations of observed to predicted values. Agreeing to Parresol [119], SEE, MPE, and MPSE statistics as model performance metrics were first utilized by Schlaegen [120] and afterwards by Lin [53].

We now give concerned formulae.

Akaike information criterion (AIC):

(A.1) $$\begin{array}{c} \text{AIC}=-2l\left(\hat{\theta }\right)+2p. \end{array}$$

Lin's concordance correlation coefficient (CCC):

(A.2) $$\begin{array}{c} \text{CCC}=\frac{2\rho \sigma_{Y}\sigma_{X}}{{\left(\mu_{X}-\mu_{Y}\right)}^{2}+\sigma_{Y}^{2}+\sigma_{Y}^{2}}, \end{array}$$

being *ρ* the Pearson's correlation coefficient. The CCC index estimates through the following:

(A.3) $$\begin{array}{c} \hat{\text{CCC}}=\frac{2S_{YX}}{{\left(\bar{Y}-\bar{X}\right)}^{2}+S_{Y}^{2}+S_{X}^{2}}, \end{array}$$

with

(A.4) $$\begin{array}{c} \bar{Y}=\frac{1}{n}\sum y_{i},\bar{X}=\frac{1}{n}\sum x_{i},\\ S_{Y}^{2}=\frac{1}{n}\sum {\left(y_{i}-\bar{y}\right)}^{2},S_{X}^{2}=\frac{1}{n}\sum {\left(x_{i}-\bar{x}\right)}^{2},\\ S_{XY}=\frac{1}{n}\sum \left(x_{i}-\bar{x}\right)\left(y_{i}-\bar{y}\right). \end{array}$$

Standard error of estimation (SEE):

(A.5) $$\begin{array}{c} \text{SEE}=\sqrt{\sum \frac{{\left(y_{i}-{\overset{\wedge }{y}}_{i}\right)}^{2}}{\left(n-p\right)}}. \end{array}$$

Mean prediction error (MPE):

(A.6) $$\begin{array}{c} \text{MPE}=t_{\alpha }\frac{\left(\text{SEE}/\bar{y}\right)}{\sqrt{n}}/\sqrt{n}\times 100. \end{array}$$

Mean percent standard error (MPSE):

(A.7) $$\begin{array}{c} \text{MPSE}=\frac{1}{n}\sum \left|\frac{\left(y_{i}-{\hat{y}}_{i}\right)}{{\hat{y}}_{i}}\right|\times 100. \end{array}$$

The AIC index permits evaluating the operation of different entrant models that fit a given data set. The model with the smallest AIC value is judged the best among competitors. The AIC index proves a settlement between the goodness-of-fit of a model and its complexity, which express through linked log-likelihood and number of parameters as a way to penalize inclusion of unnecessary ones. Since it bases on information entropy, an AIC index is frequently understood as an estimate of the information lost when a model is used to represent the process that generates the data. Lin's concordance correlation coefficient (CCC) evaluates how well one variable (*Y*) reproduces another (*X*); in other words, it represents a measure of the similarity (or agreement) between the two variables. The standard error of estimation (SEE) bears a global evaluation of goodness-of-fit of a model to observed data, since it measures the accuracy of (${\hat{y}}_{i}$) predictions produced by a fitted regression model. This index takes nonnegative values. When SEE attains its minimum value, of zero, the observed values of the response coincide with the fitted mean response function, meaning that the model displays exact reproducibility of observed values. The MPE, which is now used to determine the goodness-of-fit of a model, is a standardized version of the coefficient of variation $\text{CV}=\left(\text{SEE}/\bar{y}\right)\times 100$ expressed as a percentage, as proposed by Schlaegen [120]. The MPSE backs a measure of the average absolute relative error, expressed as a percentage. This model assessment index was recommended by Schlaegen [120] and Meyer [121] as a measure of the absolute deviation of the expected and predicted responses, relative to the size of the prediction ($\left|y_{i}-{\hat{y}}_{i}\right|{\hat{y}}_{i}$) expressed as a percentage average. For additional information on the use of the aforesaid statistics in allometric examination, the reader is referred to [122].

### B. Comparison of Models Based on the AIC Index

In this appendix, we provide an explanation to the AICA entries in Tables 2, 5, 7, 9, and 11. To do so, we abide by the notation convention (*u*, *v*) = (ln*x*, ln*y*) to understand the transformation that carries a point (*x*, *y*) in arithmetical space into one (*u*, *v*) in logscales.

In allometry, we ought to compare different models based on the AIC index, where some models are fitted in the arithmetic space and others in log scales. Whenever a model was fitted in log scales, we denote its log-likelihood through the symbol $\log_{G}\left(\hat{\theta }\right)$. If we want to compare such a model with another one fitted in the direct scales of data, we are required to transport the likelihoods and log-likelihoods from log scales to arithmetical ones, because allometric data intrinsically associate there. Particularly, we denote the statistics resulting from transferring $\log_{G}\left(\hat{\theta }\right)$ to the arithmetical space by means of the symbol $\log_{A}\left(\hat{\theta }\right)$. The conversion of concurring log-likelihood $\log_{G}\left(\hat{\theta }\right)$ into its arithmetical space equivalence $\log_{A}\left(\hat{\theta }\right)$ achieves through the relationship:

(B.1) $$\begin{array}{c} \log_{A}\left(\hat{\theta }\right)=\log_{G}\left(\hat{\theta }\right)-\overset{n}{\underset{i=1}{\sum }}v_{i}, \end{array}$$

where $\hat{\theta }$ stands for the set of estimated parameters for a given model.

Equivalently, once a model fitted in geometrical space attains an AIC value, we make it correspond to its equivalent statistics in direct scales which we denote through AICA. Necessarily, the AICA derives from the log-likelihood expressed in direct scales $\log_{A}\left(\hat{\theta }\right)$, that is,

(B.2) $$\begin{array}{c} \text{AICA}=-2\log_{A}\left(\hat{\theta }\right)+2p. \end{array}$$

We explain how Equation (A.7) derives for the case in which the *y* as a random variable is lognormally distributed with log-mean *μ* and log-deviation *σ*, that is, *y* ~ lognorm(*μ*, *σ*) .Under such an assumption, *v* is a normally distributed having mean *μ* and deviation *σ*, that is, *v* ~ *N*(*μ*, *σ*). We begin by establishing the relationship between the loglikelihoods in the arithmetic and geometric scales. Since we have assumed that *y* ~ lognorm(*μ*, *σ*), the expression for the likelihood function in arithmetic scales log_*A*_(*θ*) becomes the following:

(B.3) $$\begin{array}{c} \log_{A}\left(\theta \right)=\overset{n}{\underset{i=1}{\prod }}\frac{1}{\sqrt{2\pi }\sigma y_{i}}\exp\left[-\frac{1}{2}{\left(\frac{\log\left(y_{i}\right)-\mu }{\sigma }\right)}^{2}\right], \end{array}$$

being *θ* = (*μ*, *σ*). Correspondingly, we have that the likelihood log_*G*_(*θ*) on the geometric scales sets by the following:

(B.4) $$\begin{array}{c} \log_{G}\left(\theta \right)=\prod \frac{1}{\sqrt{2\pi }\sigma }\exp\left[-\frac{1}{2}{\left(\frac{v_{i}-\mu }{\sigma }\right)}^{2}\right]. \end{array}$$

Since *v*_*i*_ = log(*y*_*i*_), *i* = 1, 2, ⋯, *n*, then we have the following:

(B.5) $$\begin{array}{c} \log_{A}\left(\theta \right)=\overset{n}{\underset{i=1}{\prod }}\frac{1}{y_{i}}\times \overset{n}{\underset{i=1}{\prod }}\frac{1}{\sqrt{2\pi }\sigma }\exp\left[-\frac{1}{2}{\left(\frac{\log\left(y_{i}\right)-\mu }{\sigma }\right)}^{2}\right], \end{array}$$

or equivalently,

(B.6) $$\begin{array}{c} \log_{A}\left(\theta \right)=\frac{1}{\left(\prod_{i=1}^{n}y_{i}\right)}\times \overset{n}{\underset{i=1}{\prod }}\frac{1}{\sqrt{2\pi }\sigma }\exp\left[-\frac{1}{2}{\left(\frac{v_{i}-\mu }{\sigma }\right)}^{2}\right]. \end{array}$$

Therefore,

(B.7) $$\begin{array}{c} \log_{A}\left(\theta \right)=\log_{G}\left(\theta \right)\times \frac{1}{\left(\prod_{i=1}^{n}y_{i}\right)}. \end{array}$$

To obtain the relation between the log-likelihoods log_*A*_(*θ*) and log_*G*_(*θ*), we take logarithms on both sides of Equation (B.6) which yields the following:

(B.8) $$\begin{array}{c} \log_{A}\left(\theta \right)=\log\left\{\log_{A}\left(\theta \right)\right\}=\log\left\{\log_{G}\left(\theta \right)*\frac{1}{\pi y_{i}}\right\}, \end{array}$$

or equivalently

(B.9) $$\begin{array}{c} \log_{A}\left(\theta \right)=\log\left\{\log_{G}\left(\theta \right)\right\}+\log\left(\frac{1}{\pi y_{i}}\right), \end{array}$$

which finally sets the following:

(B.10) $$\begin{array}{c} \log_{A}\left(\theta \right)=\log_{G}\left(\theta \right)+\sum \log\left(y_{i}\right). \end{array}$$

## Supplementary Materials

Supplementary Materials We provide MATLAB and R codes for the related identification tasks (Computer_codes.doc). Each code description includes comments referring to the paper section it relates to and what it generates (table of fitted parameters, figure, or reproducibility index). MATLAB 2016a or later version and RStudio are required. The set of observed data (referred by means of an EHDS acronym in the manuscript) could be available from the corresponding author-provided acceptance of a fair use agreement. However, we readily include the set of processed data resulting from the MAD procedure referred on Section 3.6 (dpro.text) and the simulated data in Section 3.11 (dsim_10410.text) that allow testing the performance of the codes.
