# Supplementary material for: A Revision of the Traditional Analysis Method of Allometry to Allow Extension of the Normality-Borne Complexity of Error Structure: Examining the Adequacy of a Normal-Mixture Distribution-Driven Error Term
Source: Biomed Res Int. 2022 Sep 19;2022:8310213. doi: 10.1155/2022/8310213 (PMC9512611; doi:10.1155/2022/8310213)
Supplement: Supplementary Materials — We provide MATLAB and R codes for the related identification tasks (Computer_codes.doc). Each code description includes comments referring to the paper section it relates to and what it generates (table of fitted parameters, figure, or reproducibility index). MATLAB 2016a or later version and RStudio are required. The set of observed data (referred by means of an EHDS acronym in the manuscript) could be available from the corresponding author-provided acceptance of a fair use agreement. However, we readily include the set of processed data resulting from the MAD procedure referred on Section 3.6 (dpro.text) and the simulated data in Section 3.11 (dsim_10410.text) that allow testing the performance of the codes. [file 8310213.f1.docx]

COMPUTER CODES

Codes provided bellow associate to the fittings of models that produced the figures and tables presented through the article entitled:

A revision of the Traditional Analysis Method of Allometry to allow extension of the normality-borne complexity of error structure: Examining the adequacy of a normal-mixture distribution driven error term, by Enrique Villa-Diharce, Héctor Echavarría-Heras, Abelardo Montesinos-López and Cecilia Leal-Ramírez

The fits of the Models, Tables and Figures were produced using Matlab and R codes. Each code includes a running title referring what it generates (Table of fitting statistics, Figure or Reproducibility Index). We provide in the supplementary files the data set sustaining the simulation study of section 3.11. Upon contacting the corresponding author the set of observed data can be made available , provided acceptance of a fair use agreement .

==== INSTALLATION ====

MATLAB 2016a or later version and R o RStudio, are required

NOTE: Before running the codes in R and Matlab, it renders it necessary to change the working directory to the directory where the programs are located so that the data file required by each of them can be read correctly.

====CODES====

%...................................................................

% Section-3.1: Figure 1

%...................................................................

clear

clc

% Data set

load dobs.txt

x = dobs(:,5); % Areas

y = dobs(:,2); % Weight

% Latex configuration

set(groot,'defaultAxesTickLabelInterpreter','latex');

set(groot,'defaulttextinterpreter','latex');

set(groot,'defaultLegendInterpreter','latex');

% Figure 1a

figure;

plot(x,y,'.b');

xlabel({'$Area [mm^2]$'});

ylabel({'$Leaf Biomass [g]$'});

title({'$a$'});

% Figure 1b

figure;

plot(log(x),log(y),'.b');

xlabel({'$log(Area)$'});

ylabel({'$log(Leaf Biomass)$'});

title({'$b$'});

%...................................................................

% Section-3.1: Figure 2

%...................................................................

clear

clc

% Data set

load dobs.txt

x = dobs(:,3); % Large

y = dobs(:,2); % Weight

n = find(dobs(:,1)==1); % first month

m1_x = x(n);

m1_y = y(n);

% Latex configuration

set(groot,'defaultAxesTickLabelInterpreter','latex');

set(groot,'defaulttextinterpreter','latex');

set(groot,'defaultLegendInterpreter','latex');

% Figure 2a

figure;

plot(m1_x,m1_y,'.r');

xlabel({'$Length [mm]$'});

ylabel({'$Weight [g]$'});

title({'$a$'});

% Figure 2b

figure;

plot(log(m1_x),log(m1_y),'.r');

xlabel({'$log(Length)$'});

ylabel({'$log(Weight)$'});

title({'$b$'});

%...................................................................

% Section-3.2: Figure 3

%...................................................................

clear

clc

% Data set

load dobs_10410.txt

x = dobs_10410(:,1);

y = dobs_10410(:,2);

% Ordered data set

[i,j] = sort(x);

x = x(j);

y = y(j);

% MEM Model fit parameters

b = 1.3543e-05;

a = 1.0239e+00;

r = 5.6609e-01;

% Mean response curve

yp = b.*x.^a;

% Latex configuration

set(groot,'defaultAxesTickLabelInterpreter','latex');

set(groot,'defaulttextinterpreter','latex');

set(groot,'defaultLegendInterpreter','latex');

% Figura 3a

figure;

plot(x,y, 'oblack', x, yp, '-b');

xlabel({'$Area [mm^2]$'});

ylabel({'$Weight[g]$'});

title({'$a$'});

% Residuals

ei = y - yp;

% Figure 3b

figure;

plot(x,ei,'oblack', [0 max(x)], [0 0], '-b');

xlabel({'$Area [mm^2]$'});

ylabel({'$Residuals$'});

title({'$b$'});

% MEM Model fit data

% data produced by the R_code_to_Tables_1_and_2

load Fig_3c_qqplot_data.txt

load Fig_3c_qqlin_data.txt

% Figure 3c

figure;

hold on

plot(Fig_3c_qqplot_data(:,1),Fig_3c_qqplot_data(:,2), '.b',...

Fig_3c_qqlin_data(:,1),Fig_3c_qqlin_data(:,2),'-r');

line([0.240 0.240], [0, max(Fig_3c_qqplot_data(:,2))]);

line([3.883 3.883], [0, max(Fig_3c_qqplot_data(:,2))]);

xlabel({'$Theorical Quantiles$'});

ylabel({'$Sample Quantiles$'});

title({'$c$'});

hold off

%...................................................................

% Section-3.2: Figure 4

%...................................................................

clear

clc

% Data set

load dobs_10410.txt

x = dobs_10410(:,1);

y = dobs_10410(:,2);

% Ordered data set

[i,j] = sort(x);

x = log(x(j));

y = log(y(j));

% TAMA Model fit Parameters

b = -11.2096;

a = 1.0239;

r = 5.6609e-01;

% Mean response curve

yp = a.*x + b;

% Latex configuration

set(groot,'defaultAxesTickLabelInterpreter','latex');

set(groot,'defaulttextinterpreter','latex');

set(groot,'defaultLegendInterpreter','latex');

% Figure 4a

figure;

plot(x,y, 'oblack', x, yp, '-b');

xlabel({'$log(Area)$'});

ylabel({'$log(Weight)$'});

title({'$a$'});

% Residuals

ei = y - yp;

% Figure 4b

figure;

plot(x,ei,'oblack', [0 max(x)], [0 0], '-b');

xlabel({'$log(Area)$'});

ylabel({'$Residuals$'});

title({'$b$'});

% TAMA Model fit data

% Data produced by the R_code_to_Tables_1_and_2

load Fig_4c_qqplot_data.txt

load Fig_4c_qqlin_data.txt

% Figure 4c

figure;

plot(Fig_4c_qqplot_data(:,1),Fig_4c_qqplot_data(:,2), '.b',...

Fig_4c_qqlin_data(:,1),Fig_4c_qqlin_data(:,2),'-r');

xlabel({'$Theorical Quantiles$'});

ylabel({'$Sample Quantiles$'});

title({'$c$'});

%...................................................................

% Section-3.3: Figure 5

%...................................................................

clear

clc

% Data set

load dobs_10410.txt

x = dobs_10410(:,1);

y = dobs_10410(:,2);

% Ordered data set

[i,j] = sort(x);

x = x(j);

y = y(j);

% DNLR-BP Model fit parameters

b = 9.76e-06;

a = 1.0883;

s = 0.002557;

% Mean response curve

yp = b.*x.^a;

% Latex configuration

set(groot,'defaultAxesTickLabelInterpreter','latex');

set(groot,'defaulttextinterpreter','latex');

set(groot,'defaultLegendInterpreter','latex');

% Figure 5a

figure;

plot(x,y, 'oblack', x, yp, '-b');

xlabel({'$Area [mm^2]$'});

ylabel({'$Weight [g]$'});

title({'$a$'});

% Residuals

ei = y - yp;

% Figure 5b

figure;

plot(x,ei,'oblack', [0 max(x)], [0 0], '-b');

xlabel({'$Area [mm^2]$'});

ylabel({'$Residuals$'});

title({'$b$'});

% DNLR-BP Model fit data

% data produced by the R_code_to_Table_3

load Fig_5c_qqplot_data.txt

load Fig_5c_qqlin_data.txt

% Figure 5c

figure;

plot(Fig_5c_qqplot_data(:,1),Fig_5c_qqplot_data(:,2), '.b',...

Fig_5c_qqlin_data(:,1),Fig_5c_qqlin_data(:,2),'-r');

xlabel({'$Theorical Quantiles$'});

ylabel({'$Sample Quantiles$'});

title({'$c$'});

%...................................................................

% Section-3.4: Figure 6a, 6b and 6c

%...................................................................

clear

clc

% Datos set

load dobs_10410.txt

x = dobs_10410(:,1);

y = dobs_10410(:,2);

% Ordered data set

[i,j] = sort(x);

x = x(j);

y = y(j);

% MEM-LogLogistic Model fit parameters

b = 1.0869e-05;

a = 1.0584e+00;

r = 2.6506e-01;

% Mean response curve

yp = b.*x.^a;

% Latex configuration

set(groot,'defaultAxesTickLabelInterpreter','latex');

set(groot,'defaulttextinterpreter','latex');

set(groot,'defaultLegendInterpreter','latex');

% Figure 6a

figure;

plot(x,y, 'oblack', x, yp, '-b');

xlabel({'$Area [mm^2]$'});

ylabel({'$Weight [g]$'});

title({'$a$'});

% Residuals

ei = y - yp;

% Figure 6b

figure;

plot(x,ei,'oblack', [0 max(x)], [0 0], '-b');

xlabel({'$Area [mm^2]$'});

ylabel({'$Residuals$'});

title({'$b$'});

% MEM-LogLogistic Model fit data

% Data produced by the R_code_to_Tables_4_and_5

load Fig_6c_qqplot_data.txt

load Fig_6c_qqlin_data.txt

% Figure 6c

figure;

plot(Fig_6c_qqplot_data(:,1),Fig_6c_qqplot_data(:,2), '.b',...

Fig_6c_qqlin_data(:,1),Fig_6c_qqlin_data(:,2),'-r');

xlabel({'$Theorical Quantiles$'});

ylabel({'$Sample Quantiles$'});

title({'$c$'});

%...................................................................

% Section-3.4: Figure 6d, 6e y 6f

%...................................................................

clear

clc

% Data set

load dobs_10410.txt

x = dobs_10410(:,1);

y = dobs_10410(:,2);

% Ordered data set

[i,j] = sort(x);

x = x(j);

y = y(j);

% TAMA-Logistic Model fit parameters

b = -11.4296;

a = 1.0584;

r = 2.6506e-01;

% Latex configuration

set(groot,'defaultAxesTickLabelInterpreter','latex');

set(groot,'defaulttextinterpreter','latex');

set(groot,'defaultLegendInterpreter','latex');

% Mean response curve (arithmetic scale)

yp = exp(b).*x.^a * gamma(1+r)*gamma(1-r);

% Mean response curve (geometric scale)

u = log(x);

v = log(y);

vp = log(yp);

% Figura 6d

figure;

plot(u,v, 'oblack', u, vp, '-b');

xlabel({'$log(Area)$'});

ylabel({'$log(0Weight)$'});

title({'$d$'});

% Residuals

v_ei = v - vp;

% Figure 6e

figure;

plot(u,v_ei,'oblack', [0 max(u)], [0 0], '-b');

xlabel({'$log(Area)$'});

ylabel({'$Residuals$'});

title({'$e$'});

% TAMA-Logistic Model fit data

% data produced by the R_code_to_Tables_4_and_5

load Fig_6f_qqplot_data.txt

load Fig_6f_qqlin_data.txt

% Figure 6f

figure;

plot(Fig_6f_qqplot_data(:,1),Fig_6f_qqplot_data(:,2), '.b',...

Fig_6f_qqlin_data(:,1),Fig_6f_qqlin_data(:,2),'-r');

xlabel({'$Theorical Quantiles$'});

ylabel({'$Sample Quantiles$'});

title({'$f$'});

%...................................................................

% Section-3.5: Figure 7

%...................................................................

clear

clc

% Data set

load dobs_10410.txt

x = dobs_10410(:,1);

y = dobs_10410(:,2);

% Ordered data set

[i,j] = sort(x);

x = x(j);

y = y(j);

% DNLR-BP-Logistic Model fit parametros

b = 7.077e-06;

a = 1.129;

s = 2.702e-4;

% Mean response curve

yp = b.*x.^a;

% Latex configuration

set(groot,'defaultAxesTickLabelInterpreter','latex');

set(groot,'defaulttextinterpreter','latex');

set(groot,'defaultLegendInterpreter','latex');

% Figure 7a

figure;

plot(x,y, 'oblack', x, yp, '-b');

xlabel({'$Area [mm^2]$'});

ylabel({'$Weight [g]$'});

title({'$a$'});

% Residuals

ei = y - yp;

% Figure 7b

figure;

plot(x,ei,'oblack', [0 max(x)], [0 0], '-b');

xlabel({'$Area [mm^2]$'});

ylabel({'$Residuals$'});

title({'$b$'});

% DNLR-BP-Logistic Model fit data

% data produced by the R_code_to_Table_6

load Fig_7c_qqplot_data.txt

load Fig_7c_qqlin_data.txt

% Figure 7c

figure;

plot(Fig_7c_qqplot_data(:,1),Fig_7c_qqplot_data(:,2), '.b',...

Fig_7c_qqlin_data(:,1),Fig_7c_qqlin_data(:,2),'-r');

xlabel({'$Theorical Quantiles$'});

ylabel({'$Sample Quantiles$'});

title({'$c$'});

%...................................................................

% Section-3.6: Figure 8

%...................................................................

clear

clc

% Processed data set

load dpro.txt

y = dpro(:,1);

x = dpro(:,2);

% Ordered processed data set

[i,j] = sort(x);

x = x(j);

y = y(j);

% Logarithmic scale

lx = log(x);

ly = log(y);

% TAMA Model fit parameters

b = -11.36;

a = 1.044;

% Mean response curve

yp = a*lx + b;

% Latex configuration

set(groot,'defaultAxesTickLabelInterpreter','latex');

set(groot,'defaulttextinterpreter','latex');

set(groot,'defaultLegendInterpreter','latex');

% Figure 8a

figure;

plot(lx,ly, 'oblack', lx, yp, '-b');

xlabel({'$log(Area)$'});

ylabel({'$log(Weight)$'});

title({'$a$'})

% Residuals

ei = ly - yp;

% Figure 8b

figure;

plot(lx,ei,'oblack', [0, max(lx)], [0 0], '-b');

xlabel({'$log(Area)$'});

ylabel({'$Residuals$'});

title({'$b$'})

% Figure 8c

figure;

qqplot(ei);

title({'$c$'}); %.....................................................................

% Section-3.7: Figure 9a, 9b y 9c

%...................................................................

clear

clc

% Latex coniguration

set(groot,'defaultAxesTickLabelInterpreter','latex');

set(groot,'defaulttextinterpreter','latex');

set(groot,'defaultLegendInterpreter','latex');

% MEM-LogM2N Model fit data

load Fig_9a_disp_data.txt

load Fig_9a_fit_data.txt

% Figure 9a

figure;

plot(Fig_9a_disp_data(:,1),Fig_9a_disp_data(:,2), '.b',...

Fig_9a_fit_data(:,1),Fig_9a_fit_data(:,2),'-r');

xlabel({'$Area [mm^2]$'});

ylabel({'$Weight [g]$'});

title({'$a$'});

% MEM-LogM2N Model fit data

load Fig_9b_res_data.txt

% Figure 9b

figure;

plot(Fig_9b_res_data(:,1),Fig_9b_res_data(:,2), '.b');

xlabel({'$Area [mm^]$'});

ylabel({'$Residuals$'});

title({'$b$'});

% MEM-LogM2N Model fit data

% data produced by the R_code_to_Tables_8_and_9

load Fig_9c_qqplot_data.txt

load Fig_9c_qqlin_data.txt

% Figure 9c

figure;

plot(Fig_9c_qqplot_data(:,1),Fig_9c_qqplot_data(:,2), '.b',...

Fig_9c_qqlin_data(:,1),Fig_9c_qqlin_data(:,2),'-r');

xlabel({'$Theorical Quantiles$'});

ylabel({'$Sample Quantiles$'});

title({'$c$'});

%...................................................................

% Section-3.7: Figures 9d, 9e and 9f

%...................................................................

clear

clc

% Datos set

load dobs_10410.txt

x = dobs_10410(:,1);

y = dobs_10410(:,2);

% Ordered data set

[i,j] = sort(x);

x = x(j);

y = y(j);

% TAMA-M2N Model Parameters

lnb = -11.7704676;

a = 1.1108684;

p = 0.7914487;

s1 = 0.2614560;

s2 = 1.1918691;

% Mean response curve

% Arithmetic space

yp = exp(lnb).*x.^a;

fc = p * exp((s1^2)/2) + (1-p) * exp((s2^2)/2);

yp = fc .* yp;

% Latex configuration

set(groot,'defaultAxesTickLabelInterpreter','latex');

set(groot,'defaulttextinterpreter','latex');

set(groot,'defaultLegendInterpreter','latex');

% Mean response curve

% Geometric space

u = log(x);

v = log(y);

vp = log(yp);

% Figure 9d

figure;

plot(u,v, 'oblack', u, vp, '-b');

xlabel({'$log(Area)$'});

ylabel({'$log(Weight)$'});

title({'$d$'});

% Residuals

v_ei = v - vp;

% Figure 9e

figure;

plot(u,v_ei,'oblack', [0 max(u)], [0 0], '-b');

xlabel({'$log(Area)$'});

ylabel({'$Residuals$'});

title({'$e$'});

% TAMA-M2N Model fit data

% data produced by the R_code_to_Tables_8_and_9

load Fig_9f_qqplot_data.txt

load Fig_9f_qqlin_data.txt

% Figure 9f

figure;

plot(Fig_9f_qqplot_data(:,1),Fig_9f_qqplot_data(:,2), '.b',...

Fig_9f_qqlin_data(:,1),Fig_9f_qqlin_data(:,2),'-r');

xlabel({'$Theorical Quantiles$'});

ylabel({'$Sample Quantiles$'});

title({'$f$'});

%...................................................................

% Section-3.8: Figure 10

%...................................................................

clear

clc

% Data set

load dobs_10410.txt

x = dobs_10410(:,1);

y = dobs_10410(:,2);

[i,j] = sort(x);

x = x(j);

y = y(j);

% DNLR_M2N_BP Model fit Parameters

b = 6.3345341e-06;

a = 1.1441543e+00;

% Mean response curve

yp = b.*x.^a;

% Latex configuration

set(groot,'defaultAxesTickLabelInterpreter','latex');

set(groot,'defaulttextinterpreter','latex');

set(groot,'defaultLegendInterpreter','latex');

% Figure 10a

figure;

plot(x,y, 'oblack', x, yp, '-b');

xlabel({'$Area [mm^2]$'});

ylabel({'$Weight [g]$'});

title({'$a$'});

% Residuals

ei = y - yp;

% Figure 10b

figure;

plot(x,ei,'oblack', [0 max(x)], [0 0], '-b');

xlabel({'$Area [mm^2]$'});

ylabel({'$Residuals$'});

title({'$b$'});

% DNLR_M2N_BP Model fit data

% data produced by the R_code_to_Table_10

load Fig_10c_qqplot_data.txt

load Fig_10c_qqlin_data.txt

% Figure 10c

figure

plot(Fig_10c_qqplot_data(:,1),Fig_10c_qqplot_data(:,2), '.b',...

Fig_10c_qqlin_data(:,1),Fig_10c_qqlin_data(:,2),'-r');

xlabel({'$Theorical Quantiles$'});

ylabel({'$Sample Quantiles$'});

title({'$c$'});

%...................................................................

% Section-3.9: Figure 11

%...................................................................

clear

clc

% Data set

load dobs_10410.txt

x = dobs_10410(:,1);

y = dobs_10410(:,2);

% Ordered data set

[i,j] = sort(x);

x = x(j);

y = y(j);

% Logarithmic scale

lx = log(x);

ly = log(y);

% TAMA Poly6 Model Parameters

p6 = -11.748088;

p5 = 6.112240;

p4 = -4.426333;

p3 = 1.516275;

p2 = -0.252736;

p1 = 0.020574;

p0 = -0.0006561;

s = 0.523300;

% Mean response curve

fx = p0*lx.^6 + p1*lx.^5 + p2*lx.^4 + p3*lx.^3 + p4*lx.^2 + p5*lx + p6;

% Arithmetic scale

yp = exp(fx) * exp((s^2)/2);

% Geometric scale

u = log(x);

v = log(y);

vp = log(yp);

% Latex configuration

set(groot,'defaultAxesTickLabelInterpreter','latex');

set(groot,'defaulttextinterpreter','latex');

set(groot,'defaultLegendInterpreter','latex');

% Figure 11a

figure;

plot(u,v, 'oblack', u, vp, '-b');

xlabel({'$log(Area)$'});

ylabel({'$log(Weight)$'});

title({'$a$'});

% Residuals

v_ei = v - vp;

% Figure 11b

figure;

plot(u,v_ei,'oblack', [0 max(u)], [0 0], '-b');

xlabel({'$log(Area)$'});

ylabel({'$Residuals$'});

title({'$b$'});

% Figure 11c

figure;

qqplot(v_ei);

xlabel({'$Theoretical Quantiles$'});

ylabel({'$Sample Quantiles$'});

title({'$c$'});

#...................................................................

# Section-3.2: MEM Model, Tables 1 and 2

#...................................................................

# Libraries

library(numDeriv)

library(nlme)

library(writexl)

library(STAR)

options(digits = 8)

# Use the following instruction to changue the directory of work

# setwd("write here the directory of work")

#...................................................................

# Reading data set

Datos<-read.csv("C:dobs_10410.csv", header = TRUE)

# data on the arithmetic scale

a<-Datos$a;

w<-Datos$w;

plot(a,w)

Datos1<-data.frame(a=a,w=w)

# data on the logarithmic scale

u<-log(a);

v<-log(w);

par(mfrow=c(1,1))

plot(u,v)

#...................................................................

# MEM Model: Table 1

#...................................................................

mll_Tab2_f<-function(theta,w,a)

{

B = exp(theta[1]); alpha = exp(theta[2]); sigma = exp(theta[3])

ll = sum(dlnorm(w,log(B)+alpha*log(a),sigma,log=TRUE))

-ll

}

# Var of each component of theta=c(B,alpha,sigma)

# from the variance of theta_a=c(exp(B),exp(alpha),log(sigma))

Var_Tab2_f<-function(theta_a,Sigma)

{

g = c(exp(theta_a[1]),exp(theta_a[2]),exp(theta_a[3]))

diag(diag(g)%*%Sigma%*%diag(g))

}

A_Tab2_f<-function(w,a)

{

A0 = lm(log(w)~log(a)); Bv = coef(A0)

theta0 = c(Bv[1],log(Bv[2]),log(summary(A0)$sigma))

A=optim(theta0,mll_Tab2_f,w=w,a=a,method='BFGS',hessian=TRUE,control=list(maxit=1e4))

logLikA = -A$value; logLikG=logLikA+sum(log(w))

AICG=-2*logLikG+2*3; AICA = -2*logLikA+2*3

parest = A$par

matinf = A$hessian

matcov = solve(matinf)

theta_a = A$par

se = sqrt(Var_Tab2_f(theta_a,matcov)) #std error of estimates of theta

theta = c(exp(theta_a[1]),exp(theta_a[2]),exp(theta_a[3]))

names(theta) = c('B','alpha','sigma')

Res = w-(theta[1]*a**theta[2])#it don't make sense (split)

list(theta = theta,se=se,Hess = A$hessian,theta_a=theta_a,

loglikG=logLikG,loglikA = logLikA, AICG = AICG,AICA = AICA,

Conv = A$convergence,

Residuals = Res)

}

A_Tab2 = A_Tab2_f(w,a)

Tab2 = data.frame(Parameter = names(A_Tab2$theta),

Estimate = A_Tab2$theta,

Std.Err=A_Tab2$se,

LI=A_Tab2$theta-qnorm(0.975)*A_Tab2$se,

LS=A_Tab2$theta+qnorm(0.975)*A_Tab2$se,

t_value = A_Tab2$theta/A_Tab2$se,

logLikG = A_Tab2$loglikG,

AICG =A_Tab2$AICG,

logLikA = A_Tab2$loglikA,

AICA =A_Tab2$AICA)

Tab2$p_value = 2*pnorm(-abs(Tab2$t_value))

Tab2

#...................................................................

# TAMA Model: Table 2

#...................................................................

mll_Tab3_f<-function(theta,v,u)

{

B0 = theta[1]; alpha = exp(theta[2]); sigma = exp(theta[3])

ll = sum(dnorm(v,B0+alpha*u,sigma,log=TRUE))

-ll

}

# Var of each component of theta=c(B0,alpha,sigma)

# from the variance of theta_a=c(B0,exp(alpha),log(sigma))

Var_Tab3_f<-function(theta_a,Sigma)

{

g = c(1,exp(theta_a[2]),exp(theta_a[3]))

diag(diag(g)%*%Sigma%*%diag(g))

}

A_Tab3_f<-function(v,u)

{

A0 = lm(v~u); Bv = coef(A0)

theta0 = c(Bv[1],log(Bv[2]),log(summary(A0)$sigma))

A=optim(theta0,mll_Tab3_f,u=u,v=v,method='BFGS',hessian=TRUE,control=list(maxit=1e4))

logLikG = -A$value; logLikA=logLikG-sum(v)

AICG=-2*logLikG+2*3; AICA = -2*logLikA+2*3

parest = A$par

matinf = A$hessian

matcov = solve(matinf)

theta_a = A$par

se = sqrt(Var_Tab3_f(theta_a,matcov)) #std error of estimates of theta

theta = c(theta_a[1],exp(theta_a[2]),exp(theta_a[3]))

names(theta) = c('B0','alpha','sigma')

Res = v-(theta[1]+theta[2]*u)

list(theta = theta,se=se,Hess = A$hessian,theta_a=theta_a,

loglikG=logLikG,loglikA = logLikA, AICG = AICG,AICA = AICA,

Conv = A$convergence,

Residuals = Res)

}

A_Tab3 = A_Tab3_f(v,u)

A_Tab3$theta

A_Tab3$se

Tab3 = data.frame(Parameter = names(A_Tab3$theta),

Estimate = A_Tab3$theta,

Std.Err=A_Tab3$se,

LI=A_Tab3$theta-qnorm(0.975)*A_Tab3$se,

LS=A_Tab3$theta+qnorm(0.975)*A_Tab3$se,

t_value = A_Tab3$theta/A_Tab3$se,

logLikG = A_Tab3$loglikG,

AICG =A_Tab3$AICG,

logLikA = A_Tab3$loglikA,

AICA =A_Tab3$AICA)

Tab3$p_value = 2*pnorm(-abs(Tab3$t_value))

Tab3

# Fitted values

plot(u,v,main='a)',xlab='log(Area)',ylab='log(Weight)')

points(u,v-A_Tab3$Residuals,col=4)

plot(u,A_Tab3$Residuals,main='b)',xlab='log(Area)',ylab='Residuals')

abline(h=0,col=4)

# Scaled residuals

Res = A_Tab3$Residuals/Tab3$Estimate[Tab3$Parameter=='sigma']

qqnorm(Res,main='c)');abline(a=0,b=1)

#...................................................................

# End: MEM Model, Tables 1 and 2

#...................................................................

#...................................................................

# Section-3.3: DNLR_BP Model, Table 3

#...................................................................

# Libraries

library(numDeriv)

library(nlme)

library(writexl)

library(STAR)

options(digits = 8)

# Use the following instruction to changue the directory of work

# setwd("write here the directory of work")

#...................................................................

# Reading data set

Datos<-read.csv("C:dobs_10410.csv", header = TRUE)

# data in arithmetic scale

a<-Datos$a;

w<-Datos$w;

plot(a,w)

Datos1<-data.frame(a=a,w=w)

# logarithmic scale data

u<-log(a);

v<-log(w);

par(mfrow=c(1,1))

plot(u,v)

#...................................................................

# DNLR_BP Model: Table 3

#...................................................................

mll_Tab4_f<-function(theta,w,a)

{

B = exp(theta[1]); alpha = exp(theta[2]); sigma = exp(theta[3])

k = exp(theta[4])

sigma = sigma*(1+k*a)

ll = sum(dnorm(w,B*a**alpha,sigma,log=TRUE))

-ll

}

# Var of each component of theta=c(B,alpha,sigma)

# from the variance of theta_a=c(exp(B),exp(alpha),log(sigma))

Var_Tab4_f<-function(theta_a,Sigma)

{

g = c(exp(theta_a[1]),exp(theta_a[2]),exp(theta_a[3]),exp(theta_a[4]))

diag(diag(g)%*%Sigma%*%diag(g))

}

A_Tab4_f<-function(w,a)

{

A0 = lm(log(w)~log(a)); Bv = coef(A0)

theta0 = c(Bv[1],log(Bv[2]),log(summary(A0)$sigma),-5)

A=optim(theta0,mll_Tab4_f,w=w,a=a,method='BFGS',hessian=TRUE,control=list(maxit=1e4))

logLikA = -A$value#; logLikG=logLikG+sum(log(a))

AICA = -2*logLikA+2*4#AICG=-2*logLikG+2*3;

parest = A$par

matinf = A$hessian

matcov = solve(matinf)

theta_a = A$par

se = sqrt(Var_Tab4_f(theta_a,matcov)) #std error of estimates of theta

theta = c(exp(theta_a[1]),exp(theta_a[2]),exp(theta_a[3]),exp(theta_a[4]))

names(theta) = c('B','alpha','sigma','k')

Res = w-(theta[1]*a**theta[2])

list(theta = theta,se=se,Hess = A$hessian,theta_a=theta_a,

loglikA = logLikA,AICA = AICA,

Conv = A$convergence,

Residuals = Res)

}

A_Tab4 = A_Tab4_f(w,a)

A_Tab4$theta

A_Tab4$se

Tab4 = data.frame(Parameter = names(A_Tab4$theta),

Estimate = A_Tab4$theta,

Std.Err=A_Tab4$se,

LI=A_Tab4$theta-qnorm(0.975)*A_Tab4$se,

LS=A_Tab4$theta+qnorm(0.975)*A_Tab4$se,

t_value = A_Tab4$theta/A_Tab4$se,

#logLikG = A_Tab4$loglikG,

#AICG =A_Tab4$AICG,

logLikA = A_Tab4$loglikA,

AICA =A_Tab4$AICA)

Tab4$p_value = 2*pnorm(-abs(Tab4$t_value))

Tab4

#...................................................................

# End DNLR_BP Model, Table 3

#...................................................................

#...................................................................

# Section-3.4: MEM-Log-Logistic Model, Table 4

#...................................................................

# Libraries

library(numDeriv)

library(nlme)

library(writexl)

options(digits = 8)

# Use the following instruction to changue the directory of work

# setwd("write here the directory of work")

#...................................................................

# Reading data set

Datos<-read.csv("C:dobs_10410.csv", header = TRUE)

# data in arithmetic scale

a<-Datos$a;

w<-Datos$w;

plot(a,w)

Datos1<-data.frame(a=a,w=w)

# Logarithmic scale data

u<-log(a);

v<-log(w);

par(mfrow=c(1,1))

plot(u,v)

#...................................................................

# MEM-Log-Logistic Model: Table 4

#...................................................................

#Log-logistic density

dllogis<-function(x,mu,sigma,log=TRUE)

{

if(log)

{

log(x)+dlogis(log(x),mu,sigma,log=TRUE)

}

else

{

1/x*dlogis(log(x),mu,sigma)

}

}

mll_Tab5_f<-function(theta,w,a)

{

B = exp(theta[1]); alpha = exp(theta[2]); sigma = exp(theta[3])

#k = exp(theta[4])

#sigma = sigma*(1+k*a)

ll = sum(dllogis(w,log(B*a**alpha),sigma,log=TRUE))

-ll

}

# Var of each component of theta=c(B,alpha,sigma)

# from the variance of theta_a=c(exp(B),exp(alpha),log(sigma))

Var_Tab5_f<-function(theta_a,Sigma)

{

g = c(exp(theta_a[1]),exp(theta_a[2]),exp(theta_a[3]))#,exp(theta_a[4]))

diag(diag(g)%*%Sigma%*%diag(g))

}

A_Tab5_f<-function(w,a)

{

A0 = lm(log(w)~log(a)); Bv = coef(A0)

theta0 = c(Bv[1],log(Bv[2]),log(summary(A0)$sigma))

A=optim(theta0,mll_Tab5_f,w=w,a=a,method='BFGS',hessian=TRUE,control=list(maxit=1e4))

logLikA = -A$value#; logLikG=logLikG+sum(log(a))

AICA = -2*logLikA+2*3#AICG=-2*logLikG+2*3;

parest = A$par

matinf = A$hessian

matcov = solve(matinf)

theta_a = A$par

se = sqrt(Var_Tab5_f(theta_a,matcov)) #std error of estimates of theta

theta = c(exp(theta_a[1]),exp(theta_a[2]),exp(theta_a[3]))#,exp(theta_a[4]))

names(theta) = c('B','alpha','sigma')#,'k')

Res = w-(theta[1]*a**theta[2])#No tienen sentido (dividir)?

list(theta = theta,se=se,Hess = A$hessian,theta_a=theta_a,

loglikA = logLikA,AICA = AICA,

Conv = A$convergence,

Residuals = Res)

}

A_Tab5 = A_Tab5_f(w,a)

A_Tab5$theta

A_Tab5$se

Tab5 = data.frame(Parameter = names(A_Tab5$theta),

Estimate = A_Tab5$theta,

Std.Err=A_Tab5$se,

LI=A_Tab5$theta-qnorm(0.975)*A_Tab5$se,

LS=A_Tab5$theta+qnorm(0.975)*A_Tab5$se,

t_value = A_Tab5$theta/A_Tab5$se,

#logLikG = A_Tab4$loglikG,

#AICG =A_Tab4$AICG,

logLikA = A_Tab5$loglikA,

AICA =A_Tab5$AICA)

Tab5$p_value = 2*pnorm(-abs(Tab5$t_value))

Tab5$p_value = ifelse(Tab5$p_value<(1*10^(-30)),'<1x10^-30',Tab5$p_value)

Tab5

#...................................................................

# TAMA-Logistic Model: Table 5

#...................................................................

mll_Tab6_f<-function(theta,v,u)

{

B0 = theta[1]; alpha = exp(theta[2]); sigma = exp(theta[3])

#k = exp(theta[4])

#sigma = sigma*(1+k*a)

ll = sum(dlogis(v,B0+alpha*u,sigma,log=TRUE))

-ll

}

options(digits = 4)

# Var of each component of theta=c(B,alpha,sigma)

# from the variance of theta_a=c(B0,exp(alpha),log(sigma))

Var_Tab6_f<-function(theta_a,Sigma)

{

g = c(1,exp(theta_a[2]),exp(theta_a[3]))#,exp(theta_a[4]))

diag(diag(g)%*%Sigma%*%diag(g))

}

A_Tab6_f<-function(v,u)

{

A0 = lm(v~u); Bv = coef(A0)

theta0 = c(Bv[1],log(Bv[2]),log(summary(A0)$sigma))

A=optim(theta0,mll_Tab6_f,v=v,u=u,method='BFGS',hessian=TRUE,control=list(maxit=1e4))

logLikG = -A$value;AICG = -2*logLikG+2*3

logLikA = logLikG -sum(v); AICA = -2*logLikA+2*3

parest = A$par

matinf = A$hessian

matcov = solve(matinf)

theta_a = A$par

se = sqrt(Var_Tab6_f(theta_a,matcov)) #std error of estimates of theta

theta = c((theta_a[1]),exp(theta_a[2]),exp(theta_a[3]))#,exp(theta_a[4]))

names(theta) = c('B0','alpha','sigma')

Res = w-(theta[1]+theta[2]*u)

list(theta = theta,se=se,Hess = A$hessian,theta_a=theta_a,

logLikG = logLikG,AICG = AICG,

logLikA = logLikA,AICA=AICA,

Conv = A$convergence,

Residuals = Res)

}

A_Tab6 = A_Tab6_f(v,u)

A_Tab6$theta

A_Tab6$se

Tab6 = data.frame(Parameter = names(A_Tab6$theta),

Estimate = A_Tab6$theta,

Std.Err=A_Tab6$se,

LI=A_Tab6$theta-qnorm(0.975)*A_Tab6$se,

LS=A_Tab6$theta+qnorm(0.975)*A_Tab6$se,

t_value = A_Tab6$theta/A_Tab6$se,

logLikA = A_Tab6$logLikA,

AICA = A_Tab6$AICA,

logLikG = A_Tab6$logLikG,

AICG =A_Tab6$AICG)

Tab6$p_value = 2*pnorm(-abs(Tab6$t_value))

Tab6$p_value = ifelse(Tab6$p_value<(1*10^(-30)),'<1x10^-30',Tab6$p_value)

Tab6

#2*pnorm(-abs(Tab6$t_value))<10^-(30)

#...................................................................

# End MEM-Log-Logistic Model, Table 4

#...................................................................

#...................................................................

# Section-3.5: DNLR-BP-Logistic Model, Table 6

#...................................................................

# Libraries

rm(list=ls(all=TRUE))

library(numDeriv)

library(nlme)

library(writexl)

options(digits = 8)

# Use the following instruction to change the directory of work

# setwd("write here the directory of work")

#...................................................................

# Reading data set

Datos<-read.csv("C:dobs_10410.csv", header = TRUE)

# data in arithmetic scale

a<-Datos$a;

w<-Datos$w;

plot(a,w)

Datos1<-data.frame(a=a,w=w)

# logarithmic scale data

u<-log(a);

v<-log(w);

par(mfrow=c(1,1))

plot(u,v)

#...................................................................

# DNLR-BP-Logistic Model: Table 6

#...................................................................

mll_Tab6_f<-function(theta,w,a)

{

beta = exp(theta[1]); alpha = exp(theta[2]); sigma = exp(theta[3])

k = exp(theta[4])

sigma = sigma*(1+k*a)

ll = sum(dlogis(w,beta*a**alpha,sigma,log=TRUE))

-ll

}

options(digits = 4)

#Estimate variance for each component of

#ML of theta=c(beta,alpha,sigma,k)

#computed from the approximate variance-covariance matrix of ML of

#theta_a=c(log(beta),log(alpha),log(sigma),log(k))

Var_Tab6_f<-function(theta_a,Sigma)

{

g = c(exp(theta_a[1]),exp(theta_a[2]),exp(theta_a[3]),exp(theta_a[4]))

diag(diag(g)%*%Sigma%*%diag(g))

}

A_Tab6_f<-function(w,a)

{

A0 = lm(log(w)~log(a)); Bv = coef(A0)

theta0 = c(Bv[1],log(Bv[2]),log(summary(A0)$sigma),-5)

A=optim(theta0,mll_Tab6_f,w=w,a=a,method='BFGS',hessian=TRUE,control=list(maxit=1e4))

logLikG = -A$value;AICG = -2*logLikG+2*4

logLikA = logLikG -sum(v); AICA = -2*logLikA+2*4

parest = A$par

matinf = A$hessian

matcov = solve(matinf)

theta_a = A$par

se = sqrt(Var_Tab6_f(theta_a,matcov)) #std error of estimates of theta

theta = c(exp(theta_a[1]),exp(theta_a[2]),exp(theta_a[3]),exp(theta_a[4]))

names(theta) = c('beta','alpha','sigma','k')

Res = w-theta[1]*a**theta[2]

list(theta = theta,se=se,Hess = A$hessian,theta_a=theta_a,

logLikG = logLikG,AICG = AICG,

logLikA = logLikA,AICA=AICA,

Conv = A$convergence,

Residuals = Res)

}

options(warn=-1)

A_Tab6 = A_Tab6_f(w,a)

A_Tab6$theta

A_Tab6$se

Tab6 = data.frame(Parameter = names(A_Tab6$theta),

Estimate = A_Tab6$theta,

Std.Err=A_Tab6$se,

LI=A_Tab6$theta-qnorm(0.975)*A_Tab6$se,

LS=A_Tab6$theta+qnorm(0.975)*A_Tab6$se,

t_value = A_Tab6$theta/A_Tab6$se,

logLikA = A_Tab6$logLikA,

AICA = A_Tab6$AICA,

logLikG = A_Tab6$logLikG,

AICG =A_Tab6$AICG)

Tab6$p_value = 2*pnorm(-abs(Tab6$t_value))

Tab6$p_value = ifelse(Tab6$p_value<(2*10^(-30)),'2x10^-16',Tab6$p_value)

Tab6

#...................................................................

# End DNLR-BP-Logistic Model, Table 6

#...................................................................

#...................................................................

# Section-3.6: TAMA Model, Table 7

#...................................................................

# Libraries

rm(list=ls(all=TRUE))

library(numDeriv)

library(nlme)

library(writexl)

options(digits = 8)

# Use the following instruction to changue the directory of work

# setwd("write here the directory of work")

...................................................................

# Reading data set

Datos<-read.csv("C:dobs_10410.csv", header = TRUE)

# data in arithmetic scale

a<-Datos$a;

w<-Datos$w;

plot(a,w)

Datos1<-data.frame(a=a,w=w)

# logarithmic scale data

u<-log(a);

v<-log(w);

par(mfrow=c(1,1))

plot(u,v)

#...................................................................

# TAMA Model: Table 7

#...................................................................

mll_Tab7_f<-function(theta,v,u)

{

B0 = theta[1]; alpha = exp(theta[2]); sigma = exp(theta[3])

ll = sum(dnorm(v,B0+alpha*u,sigma,log=TRUE))

#ll = sum(dnorm((v-(B0+alpha*u))/sigma,0,1,log=TRUE)-1/2*log(sigma))

-ll

}

#Estimate variance for each component of ML of theta=c(B0,alpha,sigma)

#computed from the approximate variance-covariance matrix of ML of

#theta_a=c(B0,log(alpha),log(sigma))

Var_Tab7_f<-function(theta_a,Sigma)

{

g = c(1,exp(theta_a[2]),exp(theta_a[3]))

diag(diag(g)%*%Sigma%*%diag(g))

}

A_Tab7_f<-function(v,u)

{

A0 = lm(v~u); Bv = coef(A0)

theta0 = c(Bv[1],log(Bv[2]),log(summary(A0)$sigma))

mll_Tab7_f(theta0,v,u)

A=optim(theta0,mll_Tab7_f,v=v,u=u,method='BFGS',hessian=TRUE,control=list(maxit=1e4))

logLikG = -A$value; logLikA=logLikG-sum(v)

AICG=-2*logLikG+2*3; AICA = -2*logLikA+2*3

parest = A$par

matinf = A$hessian

matcov = solve(matinf)

theta_a = A$par

se = sqrt(Var_Tab7_f(theta_a,matcov)) #std error of estimates of theta

theta = c((theta_a[1]),exp(theta_a[2]),exp(theta_a[3]))

names(theta) = c('B','alpha','sigma')#,'k')

Res = v-(theta[1]+theta[2]*u)

list(theta = theta,se=se,Hess = A$hessian,theta_a=theta_a,

loglikG=logLikG,loglikA = logLikA, AICG = AICG,AICA = AICA,

Conv = A$convergence,

Residuals = Res)

}

A_Tab7 = A_Tab7_f(v,u)

A_Tab7$theta

A_Tab7$se

Tab7 = data.frame(Parameter = names(A_Tab7$theta),

Estimate = A_Tab7$theta,

Std.Err=A_Tab7$se,

LI=A_Tab7$theta-qnorm(0.975)*A_Tab7$se,

LS=A_Tab7$theta+qnorm(0.975)*A_Tab7$se,

t_value = A_Tab7$theta/A_Tab7$se,

logLikG = A_Tab7$loglikG,

AICG =A_Tab7$AICG,

logLikA = A_Tab7$loglikA,

AICA =A_Tab7$AICA)

Tab7$p_value = 2*pnorm(-abs(Tab7$t_value))

Tab7

Tab7$p_value = ifelse(Tab7$p_value<(1*10^(-30)),'1x10^-30',Tab7$p_value)

Tab7

#...................................................................

# End TAMA Model, Table 7

#...................................................................

#...................................................................

# Section-3.7: MEM-LogM2N Model, Table 8 and 9

#...................................................................

# Libraries

library(numDeriv)

library(nlme)

library(writexl)

library(STAR)

options(digits = 8)

# Use the following instruction to changue the directory of work

# setwd("write here the directory of work")

# Reading data set

Datos<-read.csv("C:dobs_10410.csv", header = TRUE)

# data on the arithmetic scale

a<-Datos$a;

w<-Datos$w;

plot(a,w)

Datos1<-data.frame(a=a,w=w)

# data on the logarithmic scale

u<-log(a);

v<-log(w);

par(mfrow=c(1,1))

plot(u,v)

#...................................................................

# MEM-LogM2N Model: Table 8

#...................................................................

dlnm<-function(w,mu,sigma1,sigma2,p)

{

if(p<=0.5)

{

p*dlnorm(w,mu,sigma1)+(1-p)*dlnorm(w,mu,sigma2)

}

else{

0

}

}

dlnm(1,0,1,1,.5)

mll_Tab7_f<-function(theta,w,a)

{

B = exp(theta[1]);alpha = exp(theta[2]);

sigma1 = exp(theta[3]); sigma2 = exp(theta[4])

p = plogis(theta[5])

-sum(log(dlnm(w,log(B)+alpha*log(a),sigma1,sigma2,p)))

}

# Var of each component of theta=c(B,alpha,sigma1, sigma2,p)

# from the variance of theta_a=c(log(B),log(alpha),log(sigma1),qlogis(p))

Var_Tab7_f<-function(theta_a,Sigma)

{

g = c(exp(theta_a[1]),exp(theta_a[2]),exp(theta_a[3]),exp(theta_a[4]),

dlogis(theta_a[5]))

diag(diag(g)%*%Sigma%*%diag(g))

}

A_Tab7_f<-function(w,a)

{

A0 = lm(log(w)~log(a)); Bv = coef(A0)

theta0 = c(Bv[1],log(Bv[2]),log(summary(A0)$sigma),

log(summary(A0)$sigma),qlogis(.2))

A=optim(theta0,mll_Tab7_f,w=w,a=a,method='Nelder-Mead',hessian=TRUE,control=list(maxit=1e4))

A$par

logLikA = -A$value;AICA = -2*logLikA+2*5

parest = A$par

matinf = A$hessian

matcov = solve(matinf)

theta_a = A$par

se = sqrt(Var_Tab7_f(theta_a,matcov)) #std error of estimates of theta

theta = c(exp(theta_a[1]),exp(theta_a[2]),exp(theta_a[3]),exp(theta_a[4]),

plogis(theta_a[5]))

names(theta) = c('B','alpha','sigma1','sigma2','p')#,'k')

Res = NULL

list(theta = theta,se=se,Hess = A$hessian,theta_a=theta_a,

loglikA = logLikA,AICA = AICA,

Conv = A$convergence,

Residuals = Res)

}

A_Tab7 = A_Tab7_f(w,a)

A_Tab7$theta

A_Tab7$se

Tab7 = data.frame(Parameter = names(A_Tab7$theta),

Estimate = A_Tab7$theta,

Std.Err=A_Tab7$se,

LI=A_Tab7$theta-qnorm(0.975)*A_Tab7$se,

LS=A_Tab7$theta+qnorm(0.975)*A_Tab7$se,

t_value = A_Tab7$theta/A_Tab7$se,

#logLikG = A_Tab4$loglikG,

#AICG =A_Tab4$AICG,

logLikA = A_Tab7$loglikA,

AICA =A_Tab7$AICA)

Tab7$p_value = 2*pnorm(-abs(Tab7$t_value))

Tab7

#...................................................................

# TAMA-M2N Model: Table 9

#...................................................................

dnm<-function(v,mu,sigma1,sigma2,p)

{

if(p<=0.5)

{

p*dnorm(v,mu,sigma1)+(1-p)*dnorm(v,mu,sigma2)

}

else{

0

}

}

mll_Tab8_f<-function(theta,v,u)

{

B = exp(theta[1]);alpha = exp(theta[2]);

sigma1 = exp(theta[3]); sigma2 = exp(theta[4])

p = plogis(theta[5])

-sum(log(dnm(v,log(B)+alpha*u,sigma1,sigma2,p)))

}

# Var of each component of theta=c(B,alpha,sigma1, sigma2,p)

# from the variance of theta_a=c(log(B),log(alpha),log(sigma1),qlogis(p))

Var_Tab8_f<-function(theta_a,Sigma)

{

g = c(1,exp(theta_a[2]),exp(theta_a[3]),exp(theta_a[4]),

dlogis(theta_a[5]))

diag(diag(g)%*%Sigma%*%diag(g))

}

A_Tab8_f<-function(v,u)

{

A0 = lm(v~u); Bv = coef(A0)

theta0 = c(Bv[1],log(Bv[2]),log(summary(A0)$sigma),

log(summary(A0)$sigma),qlogis(.2))

A=optim(theta0,mll_Tab8_f,v=v,u=u,method='Nelder-Mead',hessian=TRUE,control=list(maxit=1e4))

logLikG = -A$value;AICG = -2*logLikG+2*5

logLikA = logLikG-sum(v);AICA = -2*logLikA+2*5

parest = A$par

matinf = A$hessian

matcov = solve(matinf)

theta_a = A$par

se = sqrt(Var_Tab8_f(theta_a,matcov)) #std error of estimates of theta

theta = c((theta_a[1]),exp(theta_a[2]),exp(theta_a[3]),exp(theta_a[4]),

plogis(theta_a[5]))

names(theta) = c('B0','alpha','sigma1','sigma2','p')#,'k')

Res = NULL

list(theta = theta,se=se,Hess = A$hessian,theta_a=theta_a,

logLikA = logLikA,AICA = AICA,

logLikG = logLikG,AICG = AICG,

Conv = A$convergence,

Residuals = Res)

}

A_Tab8 = A_Tab8_f(v,u)

A_Tab8$theta

A_Tab8$se

Tab8 = data.frame(Parameter = names(A_Tab8$theta),

Estimate = A_Tab8$theta,

Std.Err=A_Tab8$se,

LI=A_Tab8$theta-qnorm(0.975)*A_Tab8$se,

LS=A_Tab8$theta+qnorm(0.975)*A_Tab8$se,

t_value = A_Tab8$theta/A_Tab8$se,

logLikG = A_Tab8$logLikG,

AICG =A_Tab8$AICG,

logLikA = A_Tab8$logLikA,

AICA =A_Tab8$AICA)

Tab8$p_value = 2*pnorm(-abs(Tab8$t_value))

Tab8

#...................................................................

# End MEM-LogM2N Model: Table 8 an 9

#...................................................................

#...................................................................

# Section-3.8: DNLR-BP-M2N Model, Table 10

#...................................................................

# Libraries

library(numDeriv)

library(nlme)

library(writexl)

library(STAR)

options(digits = 8)

# Use the following instruction to changue the directory of work

# setwd("write here the directory of work")

# Reading data set

Datos<-read.csv("C:dobs_10410.csv", header = TRUE)

# data on the arithmetic scale

a<-Datos$a;

w<-Datos$w;

plot(a,w)

Datos1<-data.frame(a=a,w=w)

# data on the logarithmic scale

u<-log(a);

v<-log(w);

par(mfrow=c(1,1))

plot(u,v)

#...................................................................

# DNLR -BP-M2N Model: Table 10

#...................................................................

dnm<-function(w,mu,sigma1,sigma2,p)

{

if(p<=0.5)

{

p*dnorm(w,mu,sigma1)+(1-p)*dnorm(w,mu,sigma2)

}

else{

0

}

}

mll_Tab10_f<-function(theta,w,a)

{

beta = exp(theta[1]);alpha = exp(theta[2]);

sigma1 = exp(theta[3]); sigma2 = exp(theta[4])

k = exp(theta[5]); p = plogis(theta[6])

sigma1 = sigma1*(1+a*k)

sigma2 = sigma2*(1+a*k)

-sum(log(dnm(w,beta*a**alpha,sigma1,sigma2,p)))

}

#Estimate variance for each component of ML of theta=c(beta,alpha,sigma1, sigma2,k,p)

#computed from the approximate variance-covariance matrix of ML of

#theta_a=c(log(beta),log(alpha),log(sigma1),log(sigma2),log(k),qlogis(p))

Var_Tab10_f<-function(theta_a,Sigma)

{

g = c(exp(theta_a[1]),exp(theta_a[2]),exp(theta_a[3]),exp(theta_a[4]),

exp(theta_a[5]),dlogis(theta_a[6]))

diag(diag(g)%*%Sigma%*%diag(g))

}

A_Tab10_f<-function(w,a)

{

A0 = lm(log(w)~log(a)); Bv = coef(A0)

theta0 = c(Bv[1],log(Bv[2]),log(summary(A0)$sigma),

log(summary(A0)$sigma),-5,qlogis(.2))

mll_Tab10_f(theta0,w,a)

A=optim(theta0,mll_Tab10_f,w=w,a=a,method='Nelder-Mead',hessian=TRUE,control=list(maxit=1e4))

logLikA = -A$value;AICA = -2*logLikA+2*6

parest = A$par

matinf = A$hessian

matcov = solve(matinf)

theta_a = A$par

se = sqrt(Var_Tab10_f(theta_a,matcov)) #std error of estimates of theta

se =c(sqrt(matcov[1,1]),se)

theta = c(theta_a[1],exp(theta_a[1]),exp(theta_a[2]),exp(theta_a[3]),exp(theta_a[4]),

exp(theta_a[5]),plogis(theta_a[6]))

names(theta) = c('B0','B','alpha','sigma1','sigma2','k','p')

wp = theta['B']*a**theta['alpha']

Res =(w-wp)#/(1+theta['k']*a)

list(theta = theta,se=se,Hess = A$hessian,theta_a=theta_a,

logLikA = logLikA,AICA = AICA,

Conv = A$convergence,

Residuals = Res)

}

A_Tab10 = A_Tab10_f(w,a)

plot(a,w)

theta = A_Tab10$theta

wp = w-A_Tab10$Residuals

points(a,wp,col=4)

plot(a,A_Tab10$Residuals,ylim=c(-max(abs(A_Tab10$Residuals)),max(abs(A_Tab10$Residuals))))

abline(h=0)

#Scaled residuals

Ress = A_Tab10$Residuals/(1+theta['k']*a)

plot((a),Ress,ylim=c(-max(abs(Ress)),max(abs(Ress))))

#Agregar la grÃ¡fica QQ de la mezcla contra la de los Ress

Tab10 = data.frame(Parameter = names(A_Tab10$theta),

Estimate = A_Tab10$theta,

Std.Err = A_Tab10$se,

LI=A_Tab10$theta-qnorm(0.975)*A_Tab10$se,

LS=A_Tab10$theta+qnorm(0.975)*A_Tab10$se,

t_value = A_Tab10$theta/A_Tab10$se,

logLikA = A_Tab10$logLikA,

AICA =A_Tab10$AICA)

Tab10$p_value = 2*pnorm(-abs(Tab10$t_value))

Tab10

#...................................................................

# End DNLR -BP-M2N Model, Table 10

#...................................................................

#...................................................................

# Section-3.9: TAMA-Poly(6) Model, Table 11

#...................................................................

# Libraries

library(numDeriv)

library(nlme)

library(writexl)

options(digits = 8)

# Use the following instruction to changue the directory of work

# setwd("write here the directory of work")

# Reading data set

Datos<-read.csv("C:dobs_10410.csv", header = TRUE)

# data on the arithmetic scale

a<-Datos$a;

w<-Datos$w;

plot(a,w)

Datos1<-data.frame(a=a,w=w)

# data on the logarithmic scale

u<-log(a);

v<-log(w);

par(mfrow=c(1,1))

plot(u,v)

#...................................................................

# Section 3.9 TAMA-Poly(6) Model: Table 11

#...................................................................

#Minus loglikelihood

mll_Tab9_f<-function(theta,v,u)

{

B0 = theta[1]; alpha = theta[2:7]; sigma = exp(theta[8])

X = cbind(1,u,u^2,u^3,u^4,u^5,u^6)

ll = sum(dnorm(v,c(X%*%c(B0,alpha)),sigma,log=TRUE))

-ll

}

# Var of each component of theta=c(B0,alpha,sigma)

# from the variance of theta_a=c(B0,alpha,log(sigma))

Var_Tab9_f<-function(theta_a,Sigma)

{

g = c(1,rep(1,6),exp(theta_a[8]))

diag(diag(g)%*%Sigma%*%diag(g))

}

A_Tab9_f<-function(v,u)

{

X = cbind(1,u,u^2,u^3,u^4,u^5,u^6)

A0<-lm(v~0+X); Bv<-coef(A0)

theta0<-c(Bv[1],Bv[2:7],log(summary(A0)$sigma))

A=optim(theta0,mll_Tab9_f,u=u,v=v,method='BFGS',hessian=TRUE,control=list(maxit=1e4))

logLikG<--A$value; logLikA<-logLikG-sum(v)

AICG<--2*logLikG+2*8

AICA<--2*logLikA+2*8

parest<-A$par

matinf<-A$hessian

matcov<-solve(matinf)

theta_a = A$par

se<-sqrt(Var_Tab9_f(theta_a,matcov)) #std error of estimates of theta

theta = c(theta_a[1],theta_a[2:7],exp(theta_a[8]))

names(theta) = c('B0',paste0('alpha',1:6),'sigma')

Res = c(v-X%*%theta[1:7])

list(theta = theta,se=se,Hess = A$hessian,theta_a=theta_a,

loglikG=logLikG,loglikA = logLikA, AICG = AICG,AICA = AICA,

Conv = A$convergence,

Residuals = Res)

}

A_Tab9 = A_Tab9_f(v,u)

Tab9 = data.frame(Parameter = names(A_Tab9$theta),

Estimate = A_Tab9$theta,

Std.Err=A_Tab9$se,

LI=A_Tab9$theta-qnorm(0.975)*A_Tab9$se,

LS=A_Tab9$theta+qnorm(0.975)*A_Tab9$se,

t_value = A_Tab9$theta/A_Tab9$se,

logLikG = A_Tab9$loglikG,

AICG =A_Tab9$AICG,

logLikA = A_Tab9$loglikA,

AICA =A_Tab9$AICA)

Tab9$p_value = 2*pnorm(-abs(Tab9$t_value))

Tab9

#...................................................................

# End TAMA-Poly(6) Model, Table 11

#...................................................................

%...................................................................

% Section-3.10: Table 12, Reproducibility comparisons of MEM, MEM-LogLogistic ,DNLR-BP-Logistic, MEM-LogM2N, DNLR-BP-M2N and TAMA – Poly(6) Models.

%...................................................................

clear

clc

% Data set

load dobs_10410.txt

x = dobs_10410(:,5);

y = dobs_10410(:,2);

% MEM Model fit parametros

b = 1.3543e-05;

a = 1.0239e+00;

r = 5.6609e-01;

% Mean response curve

yp = b.*x.^a;

% Average x month

[avgy, avgyp] = avgpro(dobs_10410(:,1), y, yp);

% Reproducibility indices

CCC = flin(avgy, avgyp)

findice(avgy, avgyp, 2); % SEE, MPE, MPSE

% Relative deviation

RD = abs(mean(y) - mean(yp))/mean(y)

%...................................................................

% End: Table 12, MEM Model

%...................................................................

%...................................................................

% Section-3.10: Table 12, DNLR_BP Model

%...................................................................

clear

clc

% Data set

load dobs_10410.txt

x = dobs_10410(:,5);

y = dobs_10410(:,2);

% DNLR_BP Model fit parameters

b = 9.76e-06;

a = 1.0883;

s = 0.002557;

% Mean response curve

yp = (b.*x.^a);

% Average x month

[avgy, avgyp] = avgpro(dobs_10410(:,1), y, yp);

% Reproducibility indices

CCC = flin(avgy, avgyp) % CCC

findice(avgy, avgyp, 2); % SEE, MSE, MPSE

RMS = rms(avgy-avgyp) % RD

%...................................................................

% End: Table 12, DNLR_BP Model

%...................................................................

%...................................................................

% Section-3.10: Table 12, MEM-LogLogistic Model

%...................................................................

clc

clear

% Observed data

load dobs_10410.txt

x = dobs_10410(:,5);

y = dobs_10410(:,2);

% MEM-LogLogistic Model fit parameters

b = 1.0869e-05;

a = 1.0584e+00;

r = 2.6506e-01;

% Mean response curve

yp = b.*x.^a;

% Average x month

[avgy, avgyp] = avgpro(dobs_10410(:,1), y, yp);

% Reproducibility indices

CCC = flin(avgy, avgyp)

findice(avgy, avgyp, 2); % SEE, MSE, MPSE

% Relative deviation

RD = abs(mean(y) - mean(yp))/mean(y)

%...................................................................

% End: Table 12, MEM-LogLogistic Model

%...................................................................

%...................................................................

% Section-3.10: Table 12, MEM-LogM2N Model

%...................................................................

clear

clc

% Data set

load dobs_10410.txt

x = dobs_10410(:,5);

y = dobs_10410(:,2);

% MEM-LogM2N Model fit parameters

b = 7.7446e-06;

a = 1.1106e+00;

% Mean response curve

yp = b.*x.^a;

% Average x month

[avgy, avgyp] = avgpro(dobs_10410(:,1), y, yp);

% Reproducibility indices

CCC = flin(avgy, avgyp)

findice(avgy, avgyp, 2); % SEE, MSE, MPSE

% Relative deviation

RD = abs(mean(y) - mean(yp))/mean(y)

%...................................................................

% End: Table 12, MEM-LogM2N Model

%...................................................................

%...................................................................

% Section-3.10: Table 12, DNLR-BP-M2N Model

%...................................................................

clear

clc

% Data set

load dobs_10410.txt

x = dobs_10410(:,5);

y = dobs_10410(:,2);

% DNLR -BP-M2N Model fit parametros

b = 6.3345341e-06;

a = 1.1441543e+00;

% Mean response curve

yp = (b.*x.^a);

% Average x month

[avgy, avgyp] = avgpro(dobs_10410(:,1), y, yp);

% Reproducibility indices

CCC = flin(avgy, avgyp)

findice(avgy, avgyp, 2); % SEE, MSE, MPSE

% Relative deviation

RD = abs(mean(y) - mean(yp))/mean(y)

%...................................................................

% End: Table 12, DNLR-BP-M2N Model

%...................................................................

%...................................................................

% Section-3.10: Table 12, TAMA-POLY(6)

%...................................................................

clear

clc

% Data set

load dobs_10410.txt

x = dobs_10410(:,5);

y = dobs_10410(:,2);

lx = log(x);

ly = log(y);

% TAMA-Poly (6) Model fit parameters

p6 = -11.748088;

p5 = 6.112240;

p4 = -4.42633;

p3 = 1.516275;

p2 = -0.252736;

p1 = 0.020574;

p0 = -6.561e-04;

s = 0.52330;

% Mean response curve

fx = p0*lx.^6 + p1*lx.^5 + p2*lx.^4 + p3*lx.^3 + p4*lx.^2 + p5*lx + p6;

w = exp(fx) * exp((s^2)/2);

% Average x month

M = dobs_10410(:,1);

dat = [];

for i=1:13

n = find(M == i);

wm = w(n);

wp = mean(wm);

wobs = mean(y(n));

dat = [dat; i, wobs, wp];

end

% Reproducibility indices

CCC = flin(dat(:,2), dat(:,3))

findice(dat(:,2), dat(:,3), 8); % SEE, MSE, MPSE

% Relative deviation

RD = abs(mean(y) - mean(w))/mean(y)

%...................................................................

% End: Table 12, TAMA-POLY(6)

%...................................................................

%...................................................................

% Section-3.10 (change the name of this file by avgpro.m)

%...................................................................

function [avgy, avgyp] = avgpro(M, y, yp)

% Averages per month

avgy = [];

avgyp = [];

for i = 1:13

n = find(M == i);

avgy = [avgy; mean(y(n))];

avgyp = [avgyp; mean(yp(n))];

end

%...................................................................

% End: (change the name of this file by avgpro.m)

%...................................................................

%...................................................................

% Section-3.10 (change the name of this file by findice.m)

%...................................................................

function findice(wobs, wpro, p)

% Reproducibility indexes

yi = wobs; % raw data

yp = wpro; % projected data

myi = mean(yi);

n = length(yi);

% --------------------------------------------

% SEE

% Standar Error Estimate

% --------------------------------------------

a = (yi - yp).^2;

b = (n - p);

SEE = sqrt(sum(a)/b)

% --------------------------------------------

% MPE

% Mean Prediction Error

% --------------------------------------------

talpha = 2.0;

MPE = talpha * ((SEE/myi) / sqrt(n)) * 100

% --------------------------------------------

% MPSE

% Mean Percent Standar Error

% --------------------------------------------

a = (yi - yp);

MPSE = (sum(abs(a./yp))/n)*100

%...................................................................

% End: (change the name of this file by findice.m)

%...................................................................

%...................................................................

% Section-3.10 (change the name of this file by flin.m)

%...................................................................

% CONCORDANCE CORRELATION COEFFICIENT (1989,2000)

function [ rho ] = flin(x,y)

% x = values of x

% y = Values of y

% Number of data in the samples

n = length(x);

% Sum of X and Y

sumX = sum(x);

sumY = sum(y);

% Averages of X and Y

avgX = sumX/n;

avgY = sumY/n;

% We subtract to each element of X its average avgX

Sx = x - avgX;

% We subtract to each element of Y its average avgY

Sy = y - avgY;

% We multiply Sx by Sy

SxSy = Sx.*Sy;

% Get Sxy

sumSxSy = sum(SxSy);

Sxy = sumSxSy/n;

% Get Sx2

sumSx = sum(Sx.^2);

Sx2 = sumSx / n;

% Get Sy2

sumSy = sum(Sy.^2);

Sy2 = sumSy/n;

% Get rho

rho = (2*Sxy) / (Sx2 + Sy2 + (avgX - avgY)^2);

%...................................................................

% End: (change the name of this file by flin.m)

%...................................................................

#...................................................................

# Section-3.11: Simulation study, Tables 13 and 14

#...................................................................

# Libraries

library(numDeriv)

library(nlme)

library(writexl)

options(digits = 8)

# Use the following instruction to changue the directory of work

# setwd("write here the directory of work")

# Reading data set

dobs<-read.csv("C:/Cuarentena 2022/Paper Villa et al 2022/Codigos/Simulation/dobs_10410.csv", header = TRUE)

dsim<-read.csv("C:/Cuarentena 2022/Paper Villa et al 2022/Codigos/Simulation/dsim_10410.csv", header = TRUE)

# data on the arithmetic scale

a<-dobs$a;

w<-dobs$w;

plot(a,w)

Datos1<-data.frame(a=a,w=w)

# data on the logarithmic scale

u<-log(a);

v<-log(w);

par(mfrow=c(1,1))

plot(u,v)

#...................................................................

# MEM-LogM2N Model

#...................................................................

dlnm<-function(w,mu,sigma1,sigma2,p)

{

if(p<=0.5)

{

p*dlnorm(w,mu,sigma1)+(1-p)*dlnorm(w,mu,sigma2)

}

else{

0

}

}

dlnm(1,0,1,1,.5)

mll_Tab7_f<-function(theta,w,a)

{

B = exp(theta[1]);alpha = exp(theta[2]);

sigma1 = exp(theta[3]); sigma2 = exp(theta[4])

p = plogis(theta[5])

-sum(log(dlnm(w,log(B)+alpha*log(a),sigma1,sigma2,p)))

}

# Var of each component of theta=c(B,alpha,sigma1, sigma2,p)

# from the variance of theta_a=c(log(B),log(alpha),log(sigma1),qlogis(p))

Var_Tab7_f<-function(theta_a,Sigma)

{

g = c(exp(theta_a[1]),exp(theta_a[2]),exp(theta_a[3]),exp(theta_a[4]),

dlogis(theta_a[5]))

diag(diag(g)%*%Sigma%*%diag(g))

}

A_Tab7_f<-function(w,a)

{

A0 = lm(log(w)~log(a)); Bv = coef(A0)

theta0 = c(Bv[1],log(Bv[2]),log(summary(A0)$sigma),

log(summary(A0)$sigma),qlogis(.2))

A=optim(theta0,mll_Tab7_f,w=w,a=a,method='Nelder-Mead',hessian=TRUE,control=list(maxit=1e4))

A$par

logLikA = -A$value;AICA = -2*logLikA+2*5

parest = A$par

matinf = A$hessian

matcov = solve(matinf)

theta_a = A$par

se = sqrt(Var_Tab7_f(theta_a,matcov)) #std error of estimates of theta

theta = c(exp(theta_a[1]),exp(theta_a[2]),exp(theta_a[3]),exp(theta_a[4]),

plogis(theta_a[5]))

names(theta) = c('B','alpha','sigma1','sigma2','p')#,'k')

Res = NULL

list(theta = theta,se=se,Hess = A$hessian,theta_a=theta_a,

loglikA = logLikA,AICA = AICA,

Conv = A$convergence,

Residuals = Res)

}

A_Tab7 = A_Tab7_f(w,a)

A_Tab7$theta

A_Tab7$se

Tab7 = data.frame(Parameter = names(A_Tab7$theta),

Estimate = A_Tab7$theta,

Std.Err=A_Tab7$se,

LI=A_Tab7$theta-qnorm(0.975)*A_Tab7$se,

LS=A_Tab7$theta+qnorm(0.975)*A_Tab7$se,

t_value = A_Tab7$theta/A_Tab7$se,

logLikA = A_Tab7$loglikA,

AICA =A_Tab7$AICA)

Tab7$p_value = 2*pnorm(-abs(Tab7$t_value))

Tab7

# Reference parameters

pms_ref<-c(A_Tab7$theta[[1]],A_Tab7$theta[[2]],A_Tab7$theta[[3]],A_Tab7$theta[[4]],A_Tab7$theta[[5]])

pms_ref

# Simulated data set

a<-dsim$a;

w<-dsim$w;

# Fitting simulated data set

n<-1000;

pms_list <- matrix(1:5000, ncol = 5)

for (i in 1:n)

{

c<-sample(1:10410,10,replace = FALSE)

w_sim<-w[-c]

a_sim<-a[-c]

A_sim = A_Tab7_f(w_sim,a_sim)

pms_list[i,]<-c(A_sim$theta[[1]],A_sim$theta[[2]],A_sim$theta[[3]],A_sim$theta[[4]],A_sim$theta[[5]])

print(i)

}

# Parameter means obtained from simulated data

pms_means<-colMeans(pms_list)

pms_means

# RMSE

dif<-matrix(c(pms_ref[1]-pms_list[,1], pms_ref[2]-pms_list[,2], pms_ref[3]-pms_list[,3], pms_ref[4]-pms_list[,4], pms_ref[5]-pms_list[,5]),nrow=1000,ncol=5)

dif2<-dif^2;

RMSE<-sqrt(colSums(dif2)/n)

RMSE

# Relative RMSE

relRMSE<-(RMSE*100)/pms_ref

relRMSE

#write.table(pms_list, "pms_list.txt", sep = "\t", quote = F, row.names = F)

#...................................................................

# End Simulation study

#...................................................................

%...................................................................

% Section-3.11: Table 14

%...................................................................

clear

clc

% ------------------

% Observed data set

% ------------------

load dobs_10410.txt

x_obs = dobs_10410(:,1);

y_obs = dobs_10410(:,2);

% Ordered data set

[i,j] = sort(x_obs);

x_obs = x_obs(j);

y_obs = y_obs(j);

% MEM-M2N Model Parameters

b = 7.7446e-06;

a = 1.1106e+00;

p = 2.1182e-01;

s1 = 1.1682e+00;

s2 = 2.6053e-01;

% Mean response curve

yp_obs = b.*x_obs.^a;

fc = p * exp((s1^2)/2) + (1-p) * exp((s2^2)/2);

w_obs = fc .* yp_obs;

% Reproducibility indices for observed data set

CCC_obs = flin(y_obs,w_obs)

findice(y_obs,w_obs,5)

% Relative deviation

RD_obs = abs(mean(y_obs) - mean(w_obs))/mean(y_obs)

% -------------------

% Simulated data set

% -------------------

load dsim_10410.txt

x_sim = dsim_10410(:,1);

y_sim = dsim_10410(:,2);

% MEM-M2N Model Parameters

b = 9.5647977e-06;

a = 1.1111644e+00;

p = 2.0045907e-01;

s1 = 3.2352742e-01;

s2 = 3.2352704e-01;

% Mean response curve

yp_sim = b.*x_sim.^a;

fc = p * exp((s1^2)/2) + (1-p) * exp((s2^2)/2);

w_sim = fc .* yp_sim;

% Reproducibility indices for simulated data set

CCC_sim = flin(y_obs,w_sim)

findice(y_obs,w_sim,5)

% Relative deviation

RD_sim = abs(mean(y_obs) - mean(w_sim))/mean(y_obs)

%...................................................................

% End Section-3.11: Table 14

%...................................................................

%...................................................................

% Section-3.11: generate a simulated data set

%...................................................................

clear

clc

% Datos set

load dobs_10410.txt

x = dobs_10410(:,1);

y = dobs_10410(:,2);

% Ordered data set

[i,j] = sort(x);

x = x(j);

y = y(j);

%Use Parameter values produced by the fit of the MEM-M2N Model

b = 7.7446e-06;

a = 1.1106e+00;

p = 2.1182e-01;

s1 = 1.1682e+00;

s2 = 2.6053e-01;

% Mean response curve

yp = b.*x.^a;

fc = p * exp((s1^2)/2) + (1-p) * exp((s2^2)/2);

w = fc .* yp;

figure;

plot(x,y,'.b',x,w,'-r');

xlabel('Leaf area (mm^2)');

ylabel('Leaf biomass (g)');

legend('w_i = \beta a_{i}^\alpha');

% --------------------------------------------------------------------

% Generation of random data around the reference curve (Equation 40)

% --------------------------------------------------------------------

asim = [];

wsim = [];

for i = 1 : 10410

epsilon = p .* random('norm',0,s1) + (1 - p) .* random('norm',0,s2);

wsim = [wsim; w(i) * exp(epsilon)];

asim = [asim; x(i)];

end

% dsim = [asim,wsim];

% save dsim.txt dsim -ascii

% Reference curve and replicas

figure;

plot(asim,wsim,'.b',x,w,'-r');

xlabel('Leaf area (mm^2)');

ylabel('Leaf biomass (g)');

legend('w_i(\epsilon) = \beta a_{i}^\alpha e^{(\epsilon)}', 'w_i = \beta a_{i}^\alpha');

%...................................................................

% End: generate a simulated data set

%...................................................................

%...................................................................

% Section-3.11 (change the name of this file by findices.m)

%...................................................................

function findice(wobs, wpro, p)

% Reproducibility index values

yi = wobs; % raw data

yp = wpro; % projected data

myi = mean(yi);

n = length(yi);

% --------------------------------------------

% SEE

% Standard Error Estimate

% --------------------------------------------

a = (yi - yp).^2;

b = (n - p);

SEE = sqrt(sum(a)/b)

% --------------------------------------------

% MPE

% Mean Prediction Error

% --------------------------------------------

talpha = 2.0;

MPE = talpha * ((SEE/myi) / sqrt(n)) * 100

% --------------------------------------------

% MPSE

% Mean Percent Standard Error

% --------------------------------------------

a = (yi - yp);

MPSE = (sum(abs(a./yp))/n)*100

%...................................................................

% End: (change the name of this file by findices.m)

%...................................................................
